# Multiple transcription factors directly regulate Hox gene *lin-39* expression in ventral hypodermal cells of the *C. elegans* embryo and larva, including the hypodermal fate regulators LIN-26 and ELT-6

**DOI:** 10.1186/1471-213X-14-17

**Published:** 2014-05-13

**Authors:** Wan-Ju Liu, John S Reece-Hoyes, Albertha JM Walhout, David M Eisenmann

**Affiliations:** 1 Department of Biological Sciences, University of Maryland Baltimore County, Baltimore 21250, USA; 2 Department of Biochemistry and Molecular Biology, Marlene and Stewart Greenebaum Cancer Center, University of Maryland School of Medicine, Baltimore 21201, USA; 3 Program in Systems Biology, University of Massachusetts Medical School, Worcester, MA 01605, USA

**Keywords:** *C. elegans*, Hox, Gene expression, GATA, Development, Cell fate

## Abstract

**Background:**

Hox genes encode master regulators of regional fate specification during early metazoan development. Much is known about the initiation and regulation of Hox gene expression in Drosophila and vertebrates, but less is known in the non-arthropod invertebrate model system, *C. elegans*. The *C. elegans* Hox gene *lin-39* is required for correct fate specification in the midbody region, including the Vulval Precursor Cells (VPCs). To better understand *lin-39* regulation and function, we aimed to identify transcription factors necessary for *lin-39* expression in the VPCs, and in particular sought factors that initiate *lin-39* expression in the embryo.

**Results:**

We used the yeast one-hybrid (Y1H) method to screen for factors that bound to 13 fragments from the *lin-39* region: twelve fragments contained sequences conserved between *C. elegans* and two other nematode species, while one fragment was known to drive reporter gene expression in the early embryo in cells that generate the VPCs. Sixteen transcription factors that bind to eight *lin-39* genomic fragments were identified in yeast, and we characterized several factors by verifying their physical interactions *in vitro*, and showing that reduction of their function leads to alterations in *lin-39* levels and *lin-39::GFP* reporter expression *in vivo*. Three factors, the orphan nuclear hormone receptor NHR-43, the hypodermal fate regulator LIN-26, and the GATA factor ELT-6 positively regulate *lin-39* expression in the embryonic precursors to the VPCs. In particular, ELT-6 interacts with an enhancer that drives GFP expression in the early embryo, and the ELT-6 site we identified is necessary for proper embryonic expression. These three factors, along with the factors ZTF-17, BED-3 and TBX-9, also positively regulate *lin-39* expression in the larval VPCs.

**Conclusions:**

These results significantly expand the number of factors known to directly bind and regulate *lin-39* expression, identify the first factors required for *lin-39* expression in the embryo, and hint at a positive feedback mechanism involving GATA factors that maintains *lin-39* expression in the vulval lineage. This work indicates that, as in other organisms, the regulation of Hox gene expression in *C. elegans* is complicated, redundant and robust.

## Background

Hox genes encode evolutionarily conserved homeodomain-containing transcription factors that pattern cells and tissues along the anterior–posterior body axis during metazoan development (for review, see [1,2]). Hox proteins perform this function by serving as master regulators of expression of batteries of genes that impart identity to a cell [3,4], and the precise regulation of Hox protein activity is therefore vital for proper development. Due to their central and conserved role in regional identity and fate specification during metazoan development, the mechanisms underlying the initiation, maintenance and modulation of Hox gene expression have been intensively studied [5-8]. In Drosophila, an elaborate network of maternally supplied and zygotically expressed transcription factors act to initiate Hox gene expression properly in the syncytial early embryo, while in the cellularized vertebrate embryo, secreted signaling molecules and growth factors are employed to coordinate precise Hox gene expression in time and space [9,10]. In both vertebrates and Drosophila, once Hox gene expression is initiated, it is maintained by the Trithorax and Polycomb groups of chromatin regulatory proteins [11,12], and can be further modulated by extracellular signaling pathways, Hox protein autoregulation and cross-regulation, and other mechanisms [5,13].

As in other metazoans, Hox genes are essential during the development of the nematode *Caenorhabditis elegans*[14]. *C. elegans* has only six Hox genes, present in a dispersed cluster [15,16]. Three Hox genes, *ceh-13*, *nob-1*, and *php-3*, are required for proper embryonic development [17-19]. The other three Hox genes, *lin-39*, *mab-5*, and *egl-5*, appear to be required only during post-embryonic development, however their expression begins in the embryo [20-22]. Very little is known about the initiation of Hox gene regulation in *C. elegans* in the embryo [23], although subsequent regulation of Hox gene expression in larval development by Polycomb and Trithorax-related proteins, signaling pathways, other transcription factors, microRNAs, and Hox proteins themselves, have all been noted [12,23-40]. Elucidating the mechanisms by which Hox gene expression is initiated and regulated in nematodes will broaden our understanding of this important class of developmental regulators across a larger range of animal phyla, giving us further insight into their use during the evolution of animal diversity and their function in gene regulatory networks controlling pattern formation.

Our laboratory and others have studied the function of the Hox gene *lin-39* during nematode larval development, in particular during formation of the vulva, which is part of the hermaphrodite egg-laying apparatus. Vulval development begins in the first larval stage (L1) when the twelve ventral hypodermal blast cells, P1–P12 (P cells), divide to generate posterior daughters known as Pn.p cells [41]: the central six Pn.p cells, P3.p–P8.p, become Vulval Precursor Cells (VPCs) [42]. During the third larval stage (L3) the action of Wnt, Ras and Notch extracellular signaling pathways induces the VPCs to adopt distinct cell fates in the pattern 3°-3°-2°-1°-2°-3°, where the cells P5.p - P7.p adopt 1° and 2° (vulval) fates and divide to generate 22 cells that form the vulval opening, while P3.p, P4.p and P8.p adopt the non-vulval 3° fate, which is to divide once and fuse with the surrounding syncytial hypodermis (reviewed in [43,44]). The Hox gene *lin-39* encodes a Deformed/Sex combs reduced ortholog expressed in the midbody region, including the six VPCs [20,22]. *lin-39* acts twice during vulval development. *lin-39* is first required to generate the VPCs; in *lin-39* null mutants, the VPCs fuse with the hypodermis during the L1 stage, causing a Vulvaless (Vul) phenotype [20,22]. Little is known about the regulation of *lin-39* expression at this time in development. *lin-39* is also required at the time of VPC fate specification in the L3; loss of *lin-39* activity at this time leads to defects in VPC fate specification [28,45]. At this later time, LIN-39 acts downstream of RTK/Ras and Wnt extracellular signaling pathways [26,28,46,47].

Trans-acting factors regulating *lin-39* expression have been identified previously using a variety of methods including forward and reverse genetic analysis, evolutionary conservation, and transgenic reporter analysis. Trans-acting factors regulating *lin-39* expression during vulval development include the RTK/Ras pathway transcriptional effectors LIN-1 and LIN-31 [28,46-48], the Gli family member TRA-1 which acts downstream from the sex determination pathway [37], the zinc finger protein SEM-4 [49], the novel protein LIN-25 [47], several chromatin regulators [25,32,34,48,50], and LIN-39 itself [28,47]. Direct binding to sites within the *lin-39* genomic region has been established in the case of LIN-1 (and LET-418, with which it interacts), LIN-31, LIN-39 and TRA-1 [37,46-48].

We previously undertook to identify cis-acting sites regulating *lin-39* expression [47]. Due to the large size of the *lin-39* genomic region (~28 kb) we inserted fragments of *lin-39* genomic DNA upstream of an enhancerless GFP reporter. By that method, we identified three functional elements: a 340 bp upstream fragment (JW3.9) that directs expression in P cells in the embryo and in their larval descendants including the VPCs, a 247 bp site from the first *lin-39* intron that directs expression in larval ventral cord neurons, and a 1.3 kb promoter fragment (JW5) that drives expression in P6.p at the time of vulval induction. Expression from the last element is dependent on Ras pathway function and we showed that LIN-1, LIN-31 and LIN-39 directly bind this cis-regulatory module. Using an alternative approach, Kuntz et al. used phylogenetic analysis to identify a number evolutionarily-conserved regions in the *lin-39* genomic region, and showed that several of these sites also drove expression in certain cells or tissue when inserted upstream of an enhancerless GFP reporter [51]. These approaches both require that the site in question be able to mediate proper transcription activation of the reporter gene *in vivo*, and this requirement may lead to false negative results. To circumvent this issue, here we use the yeast one-hybrid assay, in which transcription factors that directly bind to a site of interest are identified, and then the function of these factors in gene regulation *in vivo* can be assayed [52].

In our previous analysis of the *lin-39* genomic region, we identified a number of short DNA sequences in the *lin-39* region that were strongly conserved between *C. elegans* and two other nematode species ([47], Supplemental material). Starting with these evolutionarily conserved elements and the 340 bp JW3.9 fragment, we used manual [53] and robotically-assisted “enhanced Y1H” (eY1H) [54] screens to identify 16 *C. elegans* transcription factors that bind to specific *lin-39* genomic DNA fragments. Seven of these factors were characterized further to determine a role in *lin-39* regulation during vulval development. We found that the orphan nuclear receptor NHR-43, the hypodermal fate regulator LIN-26 and the GATA factor ELT-6 positively regulate *lin-39* expression in the embryo and may play a role in initiation of *lin-39* in the vulval lineage. In the larva, NHR-43, LIN-26, ELT-6, the zinc finger proteins ZTF-17 and BED-3, and the T box factor TBX-9 positively regulate *lin-39* expression in the VPCs. Interestingly, we previously showed that the adjacent GATA factor genes *egl-18* and *elt-6* are downstream targets of LIN-39 in the larva VPCs [55]. Combined with our current result that ELT-6 binds to and regulates *lin-39* expression in the embryo, this suggests that EGL-18/ELT-6 and LIN-39 may form a positive feedback loop to initiate and maintain *lin-39* gene expression during embryonic and larval life to ensure proper VPC fate specification.

## Results

### Identification of transcription factors that bind to *lin-39* genomic regions using yeast one-hybrid screens

We previously identified cis and trans-acting factors that regulate *lin-39* expression in response to RTK/Ras signaling in the larval VPCs [47]. To further understand Hox gene *lin-39* regulation in *C. elegans*, we sought to identify transcription factors that bind to and regulate *lin-39* expression, with an emphasis on 1) regulation in the vulval precursor cells P3.p - P8.p (VPCs) and their descendants in the larva, and 2) expression in the precursors to the VPCs, the P cells P3 - P8, in the embryo. We used the yeast one-hybrid approach, in which DNA sequences from the gene of interest are used as ‘bait’ to screen for factors that can bind these sequences in yeast leading to activation of reporter gene expression [53]. Previously, in addition to identifying large genomic regions that drove GFP reporter expression in *lin-39*-expressing cells, we also identified 31 short DNA elements (<50 bp) located upstream, downstream and in introns that are conserved in *lin-39* from the species *C. elegans*, *C. briggsae* and *C. remanei*[47] (Additional file 1: Figure S1). Reasoning that some of these evolutionarily conserved regions (ECRs) may be binding sites for transcription factors that regulate *lin-39* expression, we used twelve small *lin-39* genomic regions (range 150–460 bp) that each encompassed one or more ECRs in yeast one-hybrid screens (Table 1, Additional file 1: Figure S1). We also used the element JW3.9, a 340 bp fragment found 7.4 kb upstream of *lin-39* that drives GFP expression in the P cells of the embryo [47], as this fragment may bind factors responsible for initiation of *lin-39* expression (Additional file 1: Figure S1). These thirteen DNA fragments, which together represent ~ 13% of the genomic region between *lin-39* and its neighboring genes, were used as separate ‘baits’ (Table 1).

**Table 1:** **Seven transcription factors interact with *lin-39 *** **genomic DNA in yeast one-hybrid assays**

**Fragment**	**ECRs**	**Size**	**TFs bound**
**YF1**	**ECR1**	**342**	**NHR-43**
**YF2**	**ECR2**	**311**	**ALR-1**
**YF3**	**ECR4**	**253**	
**YF4**	**ECR7 - 10**	**372**	**ZTF-17, LIN-26**
**YF5**	**ECR11,12**	**298**	
**YF6**	**ECR16**	**304**	
**YF7**	**ECR17**	**158**	
**YF8**	**ECR18 - 20**	**331**	
**YF9**	**ECR21 - 23**	**257**	**TBX-9**
**YF10**	**ECR24 - 26**	**319**	**BED-3**
**YF11**	**ECR27,28**	**254**	
**YF12**	**ECR29 - 33**	**455**	
**JW3.9**	**-**	**338**	**ELT-6**

The table indicates the 13 fragments (YF1-12, JW3.9) used in yeast one-hybrid screens, the evolutionarily-conserved regions (ECRs) each contains, the size of the fragment used, and the transcription factors identified as binding to each fragment.

We used two yeast one-hybrid assay procedures. One screen was performed by transformation of the thirteen bait strains with a library of 755 plasmids that each express one *C. elegans* transcription factor fused to the activation domain from the yeast transcription factor GAL4 [53,56]. Two other screens were performed using a robotically-assisted mating assay in which the thirteen haploid bait strains were mated to a collection of 936 strains, each of which expresses a single *C. elegans* transcription factor fused to the GAL4 activation domain [54]. Factors identified as positive from the primary screens were retransformed manually and tested a second time; only factors that showed a reproducible interaction in yeast were considered true positives. In total, 18 interactions were found in yeast between 16 transcription factors and eight different fragments (site bound by each factor is shown in parenthesis): NHR-43 (YF1), ALR-1 (YF2), and ZTF-17 (YF4) were identified through the library transformation screen, while 13 factors were identified via the robotically-assisted mating screens: ODR-7, TBX-39 and TBX-40 (YF1); LIN-26 (YF4); TBX-11, TBX-39 and EGL-43 (YF7); FLH-1 and NHR-111 (YF8); TBX-9 and the protein encoded by B0238.11 (YF9); BED-3 and FLH-1 (YF10); DMD-3 and ELT-6 (JW3.9) (Additional file 2: Figure S2 and Additional file 3: Figure S3, Additional file 4: Table S2 and S3, summary in Additional file 4: Table S4).

**Figure 1:**
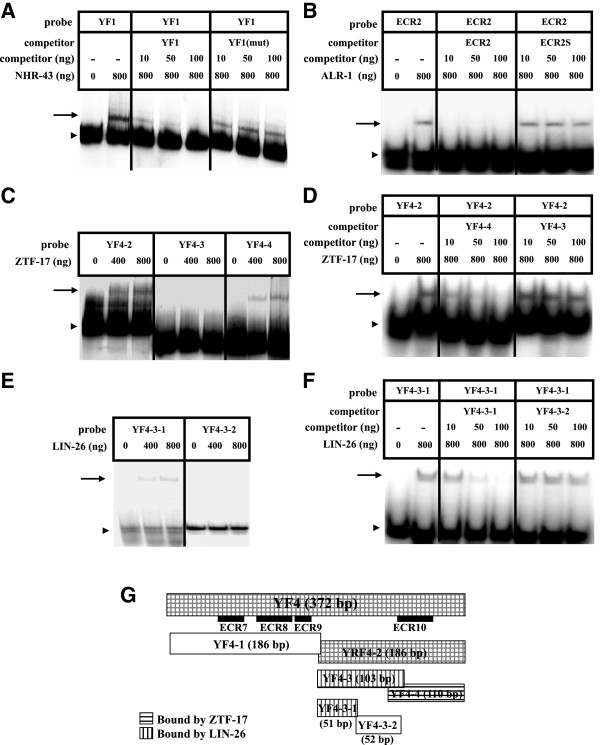
**LIN-39 regulators identified in yeast bind to *lin-39 *promoter regions *in vitro*. A - F)** Gel mobility shift assays with proteins expressed and purified from *E. coli*. Arrowhead indicates free probe; arrow indicates protein DNA complexes. For each panel, the top line identifies the labeled probe used, the bottom line shows the amount of protein added in each lane; middle lines (panels **A**, **B**, **D** and **F**) indicate the identity and amount of competitor added. **A)** NHR-43 binds to YF1 (342 bp). Lanes 3–5 contain cold wild type YF1 as competitor, while lanes 6–8 contain cold YF1 with the TGAC site mutated as competitor; **B)** ALR-1 binds to ECR2 (40 bp); this binding is competed by wild type ECR2 but not by a scrambled oligonucleotide with the same nucleotide composition (ECR2S); **C)** ZTF-17 binds to YF4-2 (186 bp) and YF4-4 (110 bp); **D)** ZTF-17 binding to YF4-2 is competed by YF4-4 but not YF4-3; **E**) LIN-26 binds to YF4-3-1 (51 bp); **F)** LIN-26 binding to YF4-3-1 is competed by YF4-3-1 (51 bp) but not YF4-3-2 (52 b); **G)** Fragment YF4 with ECRs 7–10 is shown above, with smaller subfragments diagrammed below. Shading indicates fragments that were bound by ZTF-17 and/or LIN-26 in yeast one-hybrid assays and *in vitro*.

**Figure 2:**
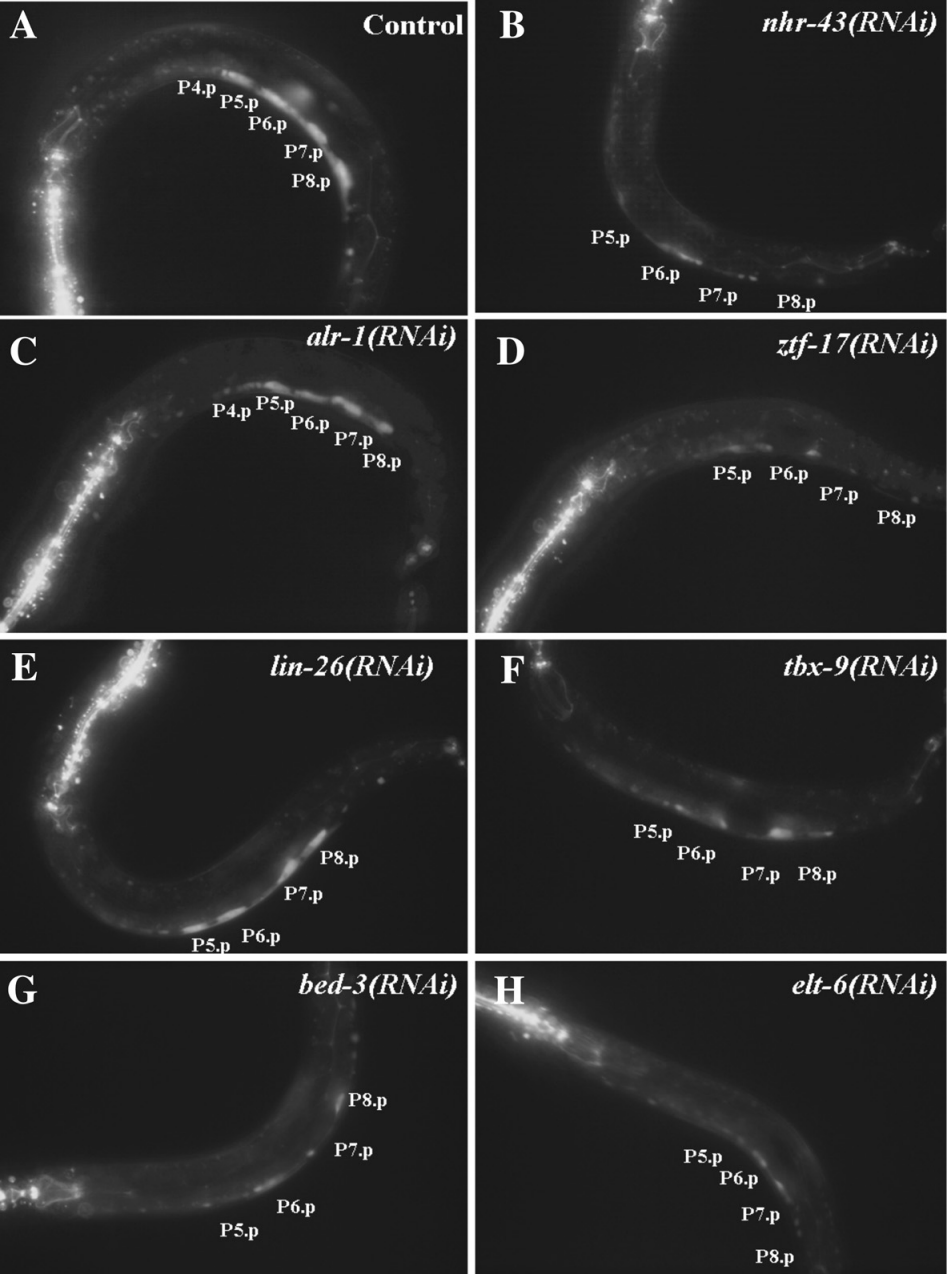
**Seven transcription factors affect *lin-39::GFP *expression in the VPCs at early L3 stage.** GFP expression in the VPCs P5.p - P8.p from *smg-1; deIS4* (*lin-39::GFP*) animals treated for RNAi of individual transcription factors identified in yeast (panels **B-H**), or given control RNAi (empty vector; panel **A**). Early L3 stage animals are shown; anterior is left, ventral is down. These animals also express *ajm-1::GFP*, which outlines cell junctions of hypodermal cells and the pharynx (bright staining in the anterior seen in most panels). RNAi for *lin-26*, *bed-3*, and *tbx-9* causes severe embryonic lethal and larva arrest phenotypes, so RNAi for these genes was performed by feeding newly-hatched L1 larvae on RNAi bacterial lawns and examining GFP expression in these same animals at the L3 stage. For all other genes, RNAi treatment was carried out on P0 animals, and their F1 progeny were examined as L3 animals. All pictures were taken under the same exposure.

**Figure 3:**
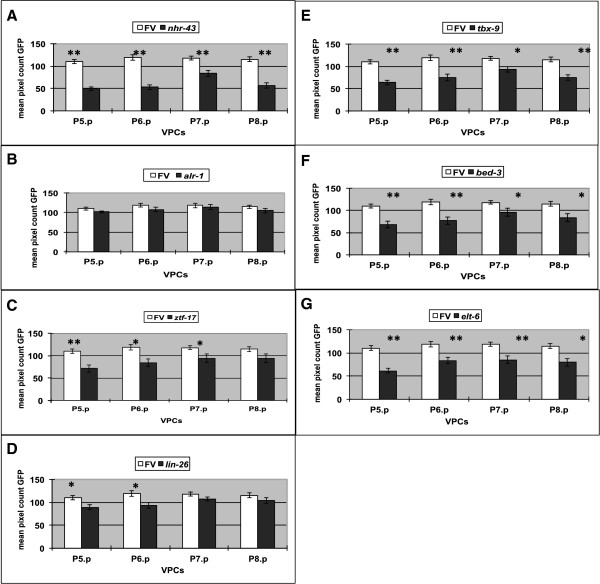
**Seven transcription factors affect *lin-39::GFP *expression in the VPCs at early L3 stage.***smg-1; deIs4*(*lin-39::GFP*) animals were treated for RNAi of individual transcription factors (panels **A–G**, dark bar), or control RNAi (panels **A–G**, light bar) as in Figure 2. Early L3 stage animals were photographed at the same exposure, and pixel counts in each VPC were determined using ImageJ (>20 animals for each strain). Bars show mean GFP pixel count in each cell with standard deviation. ‘*’ indicates *P-value* < 0.05. ‘**’ indicates *P-value* < 0.001, compared to control animals.

**Figure 4:**
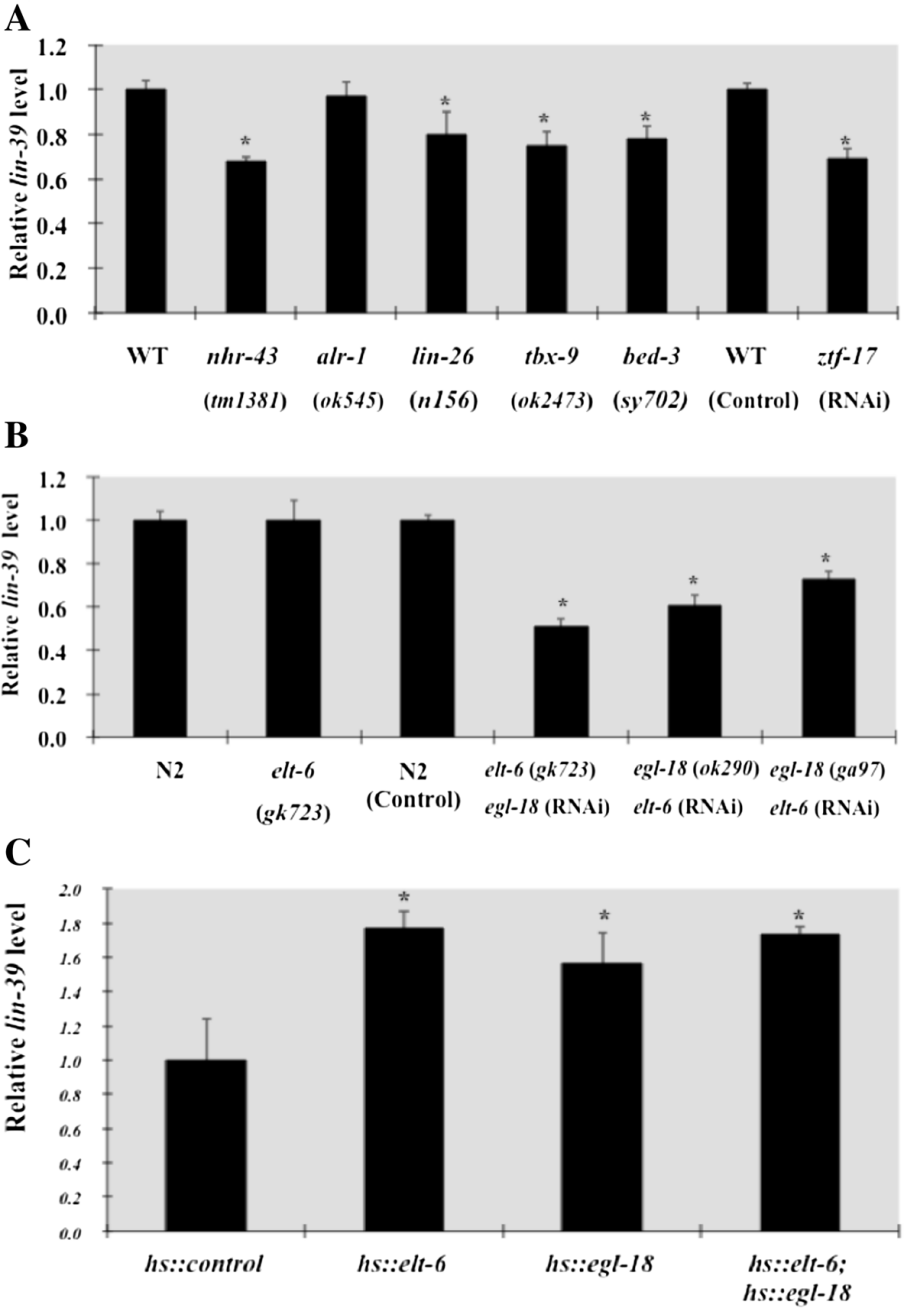
**Regulation of *lin-39 *levels by transcription factors *in vivo *(qRT-PCR)*. *A)** Decrease in *lin-39* transcript levels in *nhr-43*, *lin-26*, *tbx-9*, *bed-3* mutant and *ztf-17(RNAi)* animals. **B)** Decrease in *lin-39* transcript levels when activity of both *elt-6* and *egl-18* is reduced. **C)** Increase in *lin-39* transcript levels when either *elt-6* or *egl-18* was overexpressed from the heat shock promoter. All analyses were done on L3 stage animals. The mean values for each genotype were obtained from at least two independent experiments and normalized to the housekeeping gene, *gpd-2*, as internal standard. The data was analyzed with unpaired t-test compared to the appropriate control. ‘*’ indicates *P-value* < 0.05.

**Figure 5:**
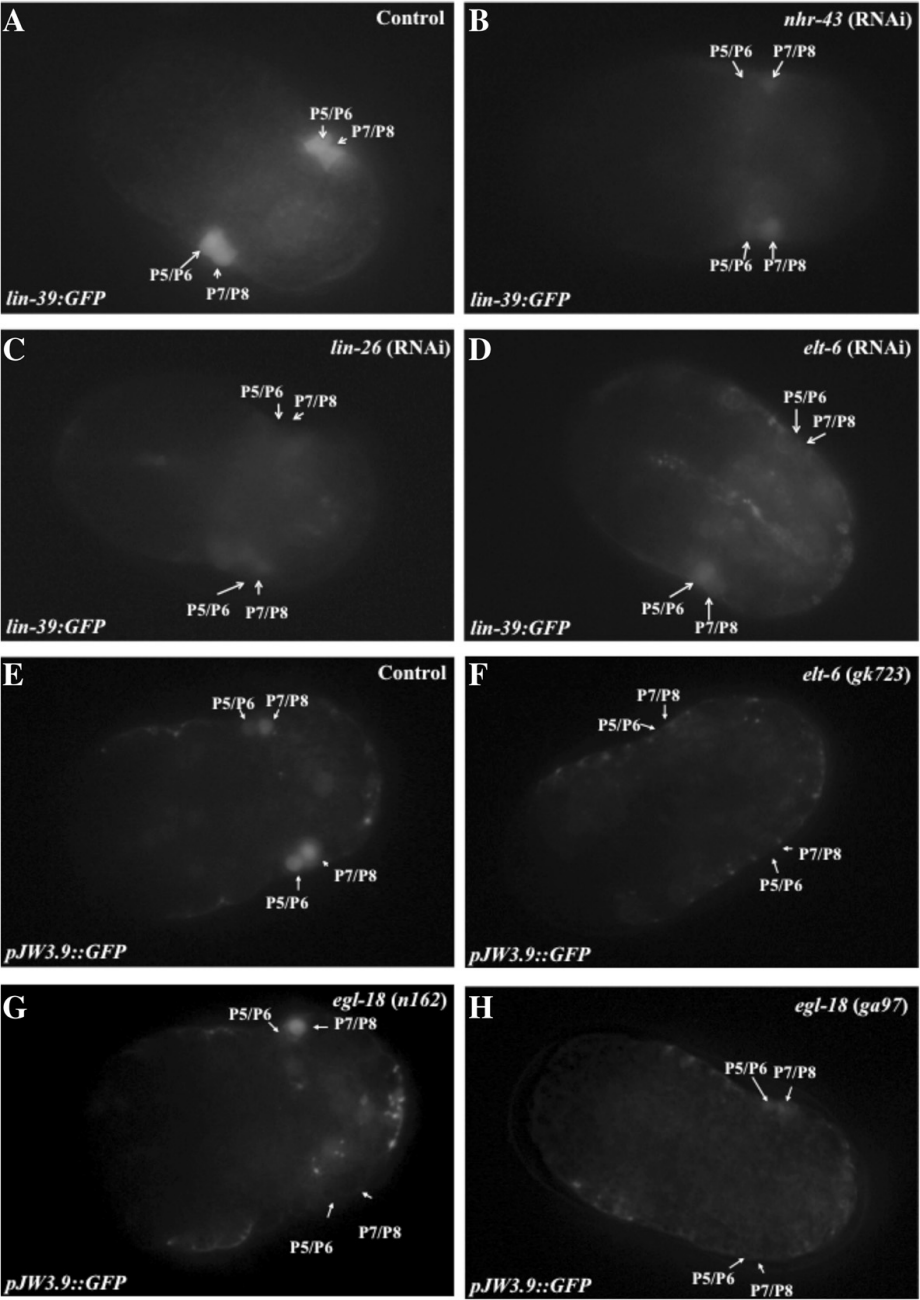
**NHR-43, LIN-26, ELT-6 and EGL-18 are necessary for *lin-39::GFP *expression in the embryo. A–D)** GFP expression in embryos derived from *smg-1; deIs4*(*lin-39::GFP*) animals treated for control RNAi (empty vector, panel **A**) or for RNAi against transcription factor genes **B)***nhr-43;***C)***lin-26* or **D)***elt-6*. **E – H)** GFP expression in embryos carrying *pJW3.9::GFP* in a wild-type background **(E)** or in animals carrying mutations in *elt-6***(F)** or *egl-18***(G and H)**. Embryos shown are at the ‘bean’ stage of embryogenesis (~360 minutes). All photos were taken under identical exposure settings. Note that before the individual P cells interdigitate at the ventral midline, the cells are referred to by their possible fates (i.e., P5/6 L and P5/6R), and there are two GFP-expressing cells on the left side and two on the right.

We describe here our characterization of seven transcription regulators (Table 1) chosen because these factors had either known expression in *lin-39*-expressing cells or a phenotype affecting a *lin-39*-regulated process [57], or because our preliminary data showed an effect on *lin-39* reporter expression *in vivo*. For each factor we carried out the following analyses. To validate the yeast interactions we expressed and purified each factor from bacteria and assayed binding to the appropriate sites *in vitro* (Figure 1, Additional file 5: Figure S6). To determine if these factors regulate *lin-39* expression *in vivo* in the vulval precursor cells during larval life, we reduced function for each transcription factor and examined *lin-39* expression using an integrated transcriptional *lin-39::GFP* reporter (*deIs4*) that contains 250 kb of genomic DNA around the *lin-39* locus [46] (Figures 2 and 3). We also examined *lin-39* expression in RNAi-treated and mutant animals at the L3 stage by qRT-PCR (Figure 4). The strain containing the integrated *lin-39::GFP* reporter was also used to examine the effects of reduced transcription factor activity on *lin-39* expression in the embryonic P cells P5 - P8, which divide in the larva to generate *lin-39*-expressing vulval precursor cells and ventral cord neurons [46] (Tables 2 and 3, Figure 5). Finally, we examined the effect of reduction of factor function in a *lin-39*-sensitized background on the fusion of the vulval precursor cells at the L2 stage (Table 4). Below we describe our results that show that six of the factors identified in the yeast one-hybrid screens regulate *lin-39* expression in the larval vulval precursor cells, while three of them also regulate *lin-39* expression in the embryo.

**Table 2:** **Decreases in *lin-39::GFP *** **expression in embryonic P cells in transcription factor RNAi animals**

**RNAi**	**N**	**% eggs with WT expression in P5-P8**
**Control**	**46**	**100%**
***nhr-43***	**40**	80%*
***alr-1***	**36**	**100%**
***ztf-17***	**36**	**100%**
***lin-26***	**36**	86%*
***tbx-9***	**37**	**100%**
***bed-3***	**36**	**94%**
***elt-6***	**51**	82%*
***egl-18***	**37**	**100%**

*smg-1*; *lin-39::GFP* animals were grown on bacteria expressing dsRNA for each transcription factor or the control vector and GFP expression in the P cells (P5 – P8) of embryos laid by these animals was examined. The percentage of animals having a wild-type intensity of GFP in all four P cells based on visual observation is indicated. ‘N’ = number of embryos scored. ‘*’ indicates P < 0.05 compared to control (Fisher’s exact test).

**Table 3:** **Decreases in *pJW3.9::GFP *** **expression in *elt-6 *** **and *egl-18 *** **mutants in embryonic P cells**

**Background**	**RNAi**	**N**	**% eggs with WT expression in P5-P8**
**Wild-type**	**-**	**50**	**98%**
**Wild-type**	**Control**	**36**	**94%**
**Wild-type**	***elt-6***	**50**	78%*
**Wild-type**	***egl-18***	**36**	**91%**
***elt-6(gk723)***	**Control**	**34**	56%*
***elt-6(gk723)***	***egl-18***	**36**	58%*
***egl-18(n162)***	**Control**	**38**	68%*
***egl-18(n162)***	***elt-6***	**36**	69%*
***egl-18(ga97)***	**Control**	**20**	60%*
***egl-18(ga97)***	***elt-6***	**24**	54%*

Animals containing *pJW3.9::GFP* in either wild type, *elt-6* mutant (*gk723*) or *egl-18* mutant (*n162* and *ga97*) backgrounds were synchronized and given the indicated RNAi treatment. *pJW3.9::GFP* expression in P5 - P8 was scored at bean stage in the embryos laid by these animals. ‘N’ = number of embryos scored. The percentage of animals having a wild-type intensity of GFP in all four P cells based on visual observation is indicated in column four. ‘*’ indicates P < 0.05 compared to control (Fisher’s exact test).

**Table 4:** **Reduction of transcription factor function in a *lin-39 *** **sensitized background affects VPC fusion**

**Strain**	**% WT VPCs**
***lin-39(n709ts) FV(RNAi)***	**96%**
***lin-39(n709ts) nhr-43(RNAi)***	**88%**
***lin-39(n709ts) alr-1(RNAi)***	72%*
***lin-39(n709ts) ztf-17(RNAi)***	**80%**
***lin-39(n709ts) lin-26(RNAi)***	65%*
***lin-39(n709ts) tbx-9(RNAi)***	72%*
***lin-39(n709ts) bed-3(RNAi)***	72%*
***lin-39(n709ts) elt-6(RNAi)***	56%*

*lin-39(n709ts); wIs79(ajm-1::GFP)* animals were grown for two generations at 25° on *E. coli* expressing dsRNA targeting the indicated gene (except for *bed-3* and *lin-26* which were grown one generation due to lethality). Larvae at the mid L2 stage (17 hrs after feeding of newly hatched L1s) were examined for *ajm-1::GFP* expression in the vulval precursor cells. Wild type animals have either 5 or 6 VPCS with *ajm-1::GFP* expression at this time (due to fusion of P3.p with hyp7). The percentage of animals showing the wild type pattern is shown (n ≥ 25 animals). ‘*’ indicates P < 0.05 compared to feeding vector (FV) control (Fisher’s exact test).

### Orphan nuclear hormone receptor NHR-43 positively regulates *lin-39* expression in the embryo and larva

The *nhr-43* gene encodes an orphan nuclear hormone receptor, and *nhr-43::GFP* reporter expression is present from late embryo to adult in hypodermis, excretory cells, posterior intestine cells, and two head neurons [58,59]. In yeast, NHR-43 bound to YF1, a 342 bp fragment located 8.6 kb upstream of *lin-39* (Additional file 1: Figure S1 and Additional file 2: Figure S2), and this binding was verified *in vitro* using NHR-43 protein purified from *E. coli* (Figure 1A). Some nuclear hormone receptors are known to bind sites containing the sequence TGAC [60], and there is a TGAC site in YF1. While the wild type YF1 sequence competed for NHR-43 binding *in vitro*, the same YF1 fragment with the TGAC site mutated no longer competed effectively (Figure 1A), suggesting that NHR-43 may bind to YF1 through this putative NHR binding site.

When RNAi was performed on worms carrying the *lin-39::GFP* transcriptional reporter to reduce *nhr-43* function, their progeny showed a decrease in the number of animals expressing wild type levels of GFP in the vulval precursor cells at the L3 stage (Figures 2B and 3A). qRT-PCR on *nhr-43(tm1381)* mutant animals [57] at the L3 stage also showed a decrease in *lin-39* expression compared to control animals (Figure 4A). We also found that fewer embryos derived from hermaphrodites treated with *nhr-43* RNAi showed *lin-39::GFP* expression in P5 - 8 compared to control animals (Figure 5B, Table 2), indicating that *nhr-43* positively regulates *lin-39::GFP* in the embryonic P cells. This embryonic defect could explain the reduction in GFP expression in the VPCs in the larva, however a decrease in *lin-39::GFP* expression was also observed if *nhr-43* RNAi was performed on L1 worms and then the same animals were observed in the L3 stage (51% of *nhr-43(L1 RNAi)* animals showed wild type expression in all VPCs versus 76% of RNAi control, p < 0.001). In summary, orphan nuclear hormone receptor NHR-43 binds to a site located far upstream of the *lin-39* initiation codon, and *nhr-43* function is required for wild type levels of *lin-39* expression in the embryo and larva in cells that will participate in vulval development.

### The Aristaless homolog ALR-1 may regulate larval *lin-39* expression

*alr-1* encodes a factor homologous to the products of the Drosophila *aristaless* and mouse *ARX* (*aristaless-related*) genes, and antibody staining and GFP reporter analysis have shown *alr-1* expression in multiple neurons and hypodermal cells from embryo to adult [61,62]. In yeast, ALR-1 bound to YF2, a 311 bp DNA fragment located 6.4 kb upstream of *lin-39* containing the evolutionarily conserved sequence ECR2 (Additional file 1: Figure S1 and Additional file 2: Figure S2). Recently, the modENCODE project identified genomic binding sites for a number of *C. elegans* transcription factors, including ALR-1, using chromatin immunoprecipitation from larval animals [40,63]. Examination of this data shows binding of ALR-1 to multiple sites in the *lin-39* promoter region, including the ECR2 site. Bacterially expressed ALR-1 protein bound to a 40 bp DNA fragment encompassing ECR2 *in vitro*, and this binding was not competed by 100 fold excess of an oligonucleotide of the same base composition and length but scrambled sequence (Figure 1B). Therefore, ALR-1 binds a site in the upstream region of *lin-39* in both *in vitro* and *in vivo* assays.

We did not observe a significant effect of *alr-1* RNAi on *lin-39* or *lin-39::GFP* expression in either the embryo or larva in the cells that will give rise to the vulva (Figures 2C, 3B, and 4A, Table 2). However, we did observe an effect of *alr-1* RNAi on a *lin-39* mediated process in the larval VPCs. During wild type development, the cells P3.p - P8.p are born in the L1 stage and because they express *lin-39* they do not fuse with the surrounding hyp7 syncytium as more anterior and posterior Pn.p cells do [20,22]. Later, in approximately 50% of animals, P3.p can fuse with hyp7 in the L2 stage [42]. Thus wild type animals have either five or six VPCs at the time of vulval induction in the late L2 stage. In *lin-39* mutant animals, or in animals in which Wnt signaling is compromised, additional VPCs are seen to adopt this Fused fate [26,28,45]. To examine the role of potential *lin-39* regulators in this process we used RNAi to reduce their function in a sensitized strain containing the hypodermal junction marker *ajm-1::GFP* and the temperature sensitive mutation *lin-39(n709ts)* and examined the fusion of the VPCs at the L2 stage as before [55]. We found an increased number of larva with VPC fusion defects in *lin-39(ts) alr-1(RNAi)* compared to *lin-39(ts)* control animals (Table 4). Therefore, although we were unable to detect an effect of loss of ALR-1 function on *lin-39* expression in the vulval cells, we did see a weak effect on a LIN-39 dependent process, leaving open its role as an *in vivo* regulator of *lin-39* expression.

### The zinc finger protein ZTF-17 positively regulates *lin-39* expression

*ztf-17* encodes a zinc finger transcription factor, and a *ztf-17::GFP* transcriptional reporter is expressed in head muscle, pharynx, and the ventral nerve cord [64]. In yeast, ZTF-17 bound to YF4, a 372 bp DNA fragment located 2 kb upstream of *lin-39* that contains ECRs 7–10 (Additional file 1: Figure S1 and Additional file 2: Figure S2). To identify a smaller ZTF-17 binding region, we divided YF4 into smaller fragments, and showed by one-hybrid assays that ZTF-17 interacts with YF4-4, a 110 bp subfragment that overlaps ECR10 (Figures 1C and E, Additional file 6: Figure S4). *in vitro* binding and competition assays with bacterially-expressed ZTF-17 protein validated this result, showing that ZTF-17 binds to fragment YF4-4, but not the adjacent fragment YF4-3 Figure 1C and D).

*ztf-17* RNAi performed on *lin-39::GFP* worms caused a decrease in the number of progeny animals showing wild type levels of *lin-39::GFP* expression in the VPCs, (Figures 2D and 3C), and qRT-PCR analysis of these *ztf-17(RNAi)* animals showed a statistically significant decrease in *lin-39* at the L3 stage (Figure 4A). *lin-39::GFP* expression in the VPC parent cells, P5 - P8, was not altered in embryos derived from *ztf-17(RNAi)* mothers (Table 2), suggesting that ZTF-17 may be a larval regulator of *lin-39* expression. In summary, *in vitro* and yeast one-hybrid analyses indicate that the zinc finger protein ZTF-17 binds to a 110 bp fragment located upstream of *lin-39*, and positively regulates *lin-39* expression in the vulval precursor cells during larval life.

### LIN-26, which is required for maintenance of hypodermal cell fates, may positively regulate *lin-39* expression in the embryo and larva

*lin-26* encodes a zinc-finger protein which is expressed in all hypodermal cells based on antibody staining [65,66]. In *lin-26* mutants, cell fate transformations from hypodermal to neuronal fate occur in many cells, including the VPCs [65,66]. LIN-26 also bound to YF4 in the yeast one-hybrid assay (Additional file 1: Figure S1 and Additional file 7: Figure S5), and further analysis showed an interaction with YF4-3, a 103 bp DNA fragment that is distinct from the fragment bound by ZTF-17 (Figure 1G, Additional file 5: Figure S5). We divided fragment YF4-3 into two smaller fragments, and *in vitro* DNA binding and competition experiments showed that LIN-26 bound to YF4-3-1, a 51 bp DNA fragment that does not contain an evolutionarily conserved region (Figure 1E and F).

To assay *lin-26* regulation of *lin-39* expression *in vivo*, *lin-26* RNAi was performed on newly hatched *lin-39::GFP* larvae and GFP levels in VPCs P5.p-P8.p were examined at the L3 stage in the same animals; this ‘L1 feeding’ was performed because of the embryonic lethal phenotype caused by *lin-26* RNAi. *lin-26* RNAi caused a weak but significant decrease in expression of *lin-39::GFP* in P5.p and P6.p at the L3 stage (Figures 2E and 3D), and qRT-PCR performed on *lin-26(n156)* mutant animals [65] also showed a small but significant decrease in *lin-39* at the early L3 stage (Figure 4A). Finally, we investigated *lin-26* regulation of *lin-39* in the embryo, and found that fewer embryos expressed *lin-39::GFP* in the P cells from mothers treated with *lin-26* RNAi, compared to control embryos (Figure 5C; Table 2). Consistent with effects on *lin-39* expression, *lin-26(RNAi)* performed on newly hatched L1 larvae in a sensitized *lin-39* background caused a defect in VPC fusion at the L2 (Table 4). Thus, we have identified a binding site in the *lin-39* upstream region for the general hypodermal transcription factor LIN-26, and our results suggest that LIN-26 acts to positively regulate *lin-39* expression in both the embryo and larva.

### The T-box protein TBX-9 binds in *lin-39* intron 2 and positively regulates larval *lin-39* expression in the VPCs

*tbx-9* encodes a T-box transcription factor, and a *tbx-9::GFP* translational reporter is expressed in lateral and ventral hypodermal cells, intestine and muscle [67]. *tbx-9* mutants have a disorganized body shape beginning in the embryo, which was attributed to defects in hypodermal cells and body wall muscles [67]. In yeast, TBX-9 bound to YF9, a 257 bp DNA fragment located in the large second intron of *lin-39* that contains ECRs 21–23 (Additional file 1: Figure S1 and Additional file 2: Figure S2), and this binding was recapitulated *in vitro* with purified TBX-9 protein (Additional file 5: Figure S6). We divided the YF9 fragment into four smaller fragments (A-D), and found that bacterially purified TBX-9 bound best to the 79 bp subfragment C, and that this fragment could also compete for binding of TBX-9 to YF9 (Additional file 5: Figure S6).

To test TBX-9 as a regulator of *lin-39 in vivo*, *tbx-9* RNAi was performed on L1 *lin-39::GFP* worms, and we observed a decrease in the number of animals with wild type levels of GFP expression in P5.p-P8.p in the same animals at the L3 stage (Figures 2F and 3E). qRT-PCR analysis on *tbx-9(ok2473)* mutant animals [57] also showed a decrease in *lin-39* levels compared to wild type L3 stage worms (Figure 4A), and *tbx-9(RNAi)* performed in a sensitized *lin-39* background caused a defect in VPC fusion at the L2 (Table 4). *tbx-9* RNAi treatment did not effect *lin-39::GFP* expression in the cells P5-P8 in the embryo (Table 2). In summary, we found that TBX-9 may bind multiple sites within a 257 bp fragment from *lin-39* intron 2, and TBX-9 acts as a positive regulator of *lin-39* expression in the VPCs in the larva.

### The Zinc-finger protein BED-3 binds to site in *lin-39* intron 2 and positively regulates *lin-39* expression

*bed-3* encodes a BED zinc-finger protein that is expressed in most hypodermal cells, including the seam cells and the progeny of the VPCs at the time of L3/L4 molt [68]. In *bed-3* mutants, the granddaughters of P5.p, P6.p and P7.p often fail to divide, which suggested that BED-3 acts late during vulval induction in the terminal divisions of the induced VPCs [68]. In yeast, BED-3 bound YF10, a 319 base pair fragment from the second *lin-39* intron that contains ECRs 24–26 (Additional file 1: Figure S1 and Additional file 2: Figure S2). Bacterially-expressed BED-3 protein bound to YF10 *in vitro*, however, we found that BED-3 protein also bound to several other unrelated DNA fragments, suggesting the purified BED-3 protein may show non-specific binding *in vitro* (data not shown).

Despite our inability to validate the BED-3 binding result *in vitro*, we observed that *bed-3* RNAi caused a strong decrease in the number of animals with wild type levels of *lin-39::GFP* expression in P5.p-P8.p at the L3 stage (Figures 2G and 3F), and this decrease was also seen by qRT-PCR analysis on *bed-3(sy702)* L3 larvae (Figure 4A). Consistent with the decrease in *lin-39* expression *in vivo*, *bed-3(RNAi)* caused a defect in VPC fusion at the L2 in a sensitized *lin-39* background (Table 4). However *lin-39::GFP* expression did not change in embryos derived from mothers treated for *bed-3(RNAi)* (Table 2). Therefore, although we could not localize a binding site for BED-3 beyond the *lin-39* intron 2 fragment used in the yeast screen, our *in vivo* data indicate that BED-3 is likely to function as a positive regulator of *lin-39* expression in the vulval precursor cells in the larva.

### The GATA factor ELT-6 binds to a *lin-39* enhancer that directs expression in the P cells in the embryo

**Figure 6:**
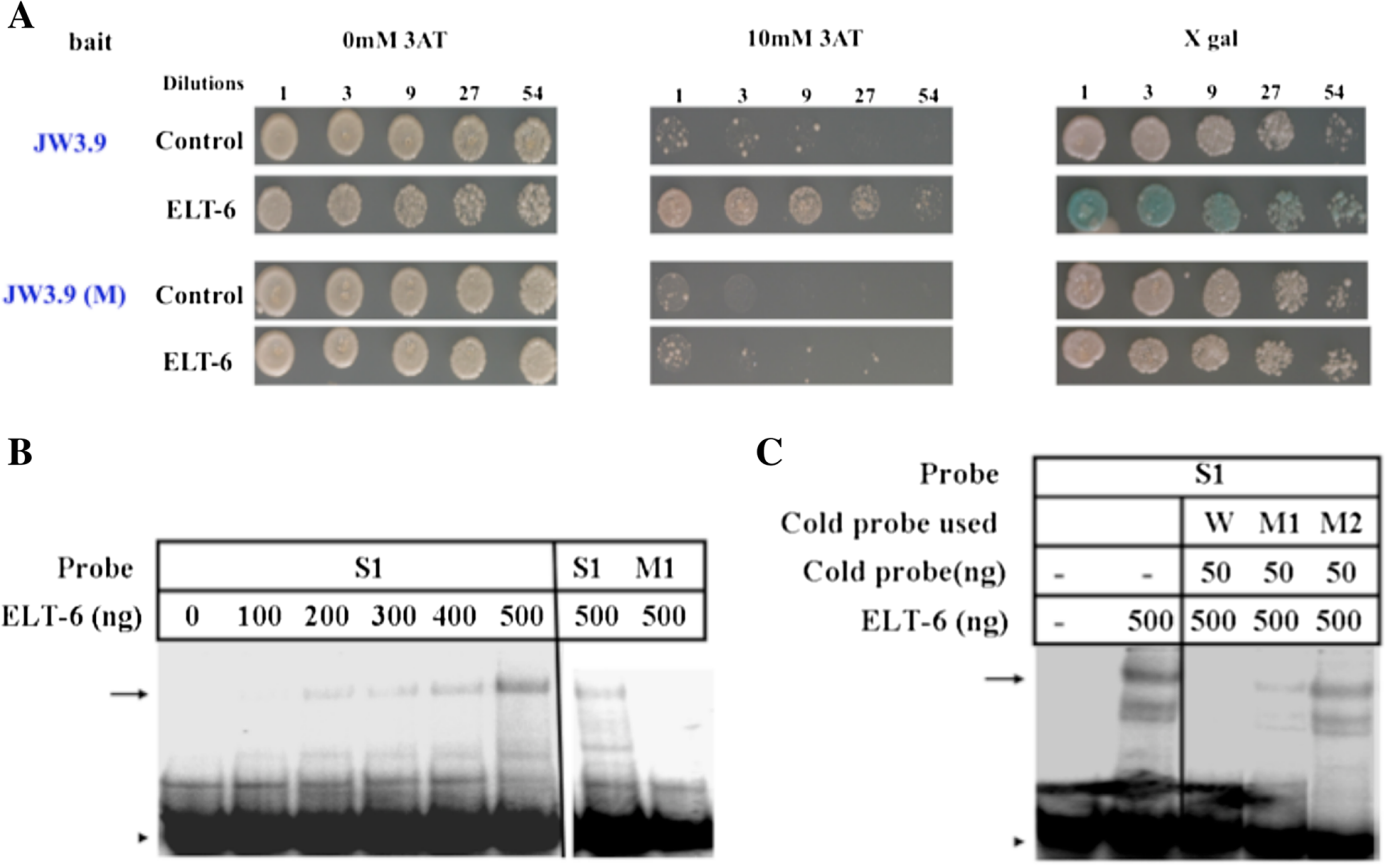
**ELT-6 interacts with *pJW3.9 *through a GATA binding site. A)** Yeast strains containing *HIS3* and *lacZ* reporters transformed with empty vector (Control) or ELT-6::GAL4AD plasmids (ELT-6) were diluted and replica plated to control (left), 10 mM 3aminotriazole (3AT, middle), and XGal (right) plates. Reporters had inserts of either JW3.9 (top) or JW3.9 in which GATA binding site S1 was mutated from TGATAA to GGTACC (‘Mut’ bottom). Mutation of the GATA site abolishes the interaction with ELT-6 based on lack of growth on 3AT and lack of blue color on XGal.; **B)** Increasing concentrations of ELT-6 interact with a 40 bp fragment around GATA site S1 (lanes 2-6). Mutation of the GATA site S1 from TGATAA to GGTACC (M1) abolishes interaction with ELT-6 (lane 8); **C)** 50 ng of cold wild type site S1 (W) can compete effectively for binding of ELT-6 to S1 (compare lanes 2 and 3). Mutation of GATA site S1 (M1) reduces but does not abolish the ability to compete (lane 4). Mutation of both site S1 and a second GATA site on the oligonucleotide (M2) drastically reduces the ability of the oligonucleotide to compete for ELT-6 binding (lane 5).

We previously described a 340 bp cis-regulatory element from *lin-39* that is sufficient to drive GFP expression in the embryo in P5-P8, cells which divide to generate *lin-39* expressing VPCs and neuroblasts in the ventral midbody region (construct *pJW3.9*, [47]). A 24 bp sequence (S1) in the *pJW3.9* enhancer is conserved between *C. elegans* and *C. briggsae*, and mutation of this site abolished embryonic expression from the *pJW3.9* reporter [47]. Site S1 contains the sequence TGATAA, a predicted binding site for a GATA family transcription factor, which prefer the motif WGATAR [69]. Intriguingly, we found that the transcription factor ELT-6 bound to the *pJW3.9* enhancer fragment in our yeast screen (Additional file 1: Figure S1 and Additional file 2: Figure S2). *elt-6* encodes a 367 amino acid GATA transcription factor expressed in certain neurons and hypodermal cells, particularly the seam cells and VPCs [55,70]. We performed directed yeast one-hybrid assays with eight other *C. elegans* GATA factors and found that ELT-6 was the only GATA factor that interacted with the *pJW3.9* enhancer fragment in yeast (Additional file 4: Table S5). When the GATA site in *pJW3.9* was mutated in the yeast reporters, ELT-6 no longer interacted with the DNA fragment in yeast (Figure 6A). ELT-6 protein purified from *E. coli* bound the S1 site *in vitro*, but did not bind when the GATA sequence was mutated (Figure 6B). ELT-6 binding to S1 was abolished when competed with excess wild type cold S1 probe, but when the S1 GATA site was mutated, the resulting oligonucleotide (M1) competed less well for ELT-6/S1 binding (Figure 6C). When a second GATA site at the edge of the 40 bp oligonucleotide was also mutated, the ability of the mutated oligonucleotide (M2) to compete was greatly reduced (Figure 6C).

To examine regulation of *lin-39* expression by ELT-6 in the embryo, we assayed expression of a reporter construct containing the wild type 340 bp element, *pJW3.9::GFP*, in *elt-6*(*gk723*) mutant animals. *gk723* is an allele with 457 bp deletion covering the first intron and second exon of *elt-6* and is a presumed null mutation [57]. Only 56% of embryos from *elt-6*(*gk723*) mutant animals displayed a wild type pattern of *pJW3.9::GFP* expression, compared to 94% of control embryos (Figure 5F, Table 3). A decrease in the penetrance of expression was also seen in the embryos derived from *pJW3.9::GFP* mothers treated with *elt-6* RNAi (78%; Table 3). We also examined the effect of reduction of *elt-6* function on embryonic expression of the large *lin-39*::*GFP* reporter that we used to assay other transcription factors. When *elt-6* RNAi was performed on *lin-39*::*GFP* hermaphrodites, only 82% of embryos showed the wild type level of GFP expression in P5- P8 (Figure 5D, Table 2). Taken together, these results indicate that the GATA factor ELT-6 is necessary for proper expression of *lin-39* in P5-P8 in the embryo, most likely via binding to the conserved GATA site in the *pJW3.9* enhancer, which was previously shown to be necessary for enhancer driven GFP expression in the embryonic P cells [47]).

### The GATA factor EGL-18 also regulates *lin-39* expression in the P cells in the embryo

**Figure 7:**
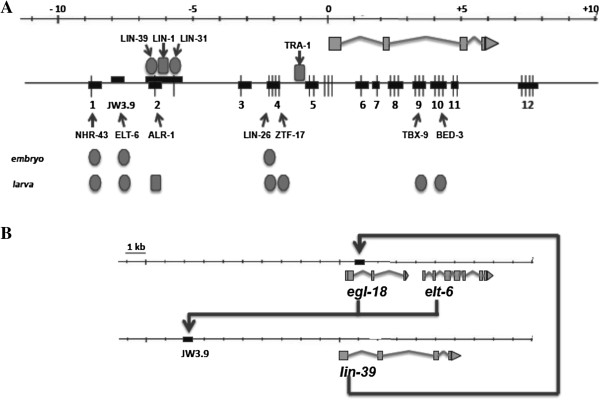
**Direct transcriptional regulators of *lin-39 *in the embryo and larva. A)** Horizontal lines represent 20 kb of genomic DNA surrounding the *lin-39* locus. The *lin-39* transcript is shown below the top line, with boxes representing exons. The next horizontal line shows evolutionarily-conserved regions (ECRs; thin vertical lines), the PCR fragments used in the yeast one hybrid assays containing the ECRs (boxes labeled 1–12), and two fragments (*pJW3.9* shown, JW5 unlabeled) identified previously using an enhancerless GFP assay [47]. Transcription factors that bind the *lin-39* gene are shown above the line (previously reported) or below the line (reported in this work). **B)** Model for positive feedback loop between *egl-18*/*elt-6* and *lin-39*. EGL-18 and ELT-6 act via the GATA site in enhancer *pJW3.9* to facilitate initiation of *lin-39* expression in the embryo, and then LIN-39 acts to positively regulate *egl-18*/*elt-6* expression via the Hox/Pbx binding site in the intron of *egl-18*[55].

The *elt-6* open reading frame begins less than 600 bp downstream from the end of another GATA factor gene, *egl-18* (see Figure 7B), and these two genes are transcribed dicistronically in some tissues [55,70]. The DNA binding domains of EGL-18 and ELT-6 are similar, and the two genes show genetic redundancy during fate specification of the hypodermal seam cells and VPCs [55,70,71]. In particular, reduction of function for both *elt-6* and *egl-18* in the larva causes the VPCs to adopt inappropriate cell fates and fuse with the hypodermal syncytium [55], a phenotype also seen with reduction of *lin-39* function [28,45].

Although EGL-18 did not bind to the 340 bp *pJW3.9* fragment in yeast assays (Additional file 4: Table S5), the known functional redundancy of *elt-6* and *egl-18* and their dicistronic expression in hypodermal cells led us to test for a role for *egl-18* in the regulation of *lin-39* expression. When the *pJW3.9::GFP* reporter was moved into two different *egl-18* mutant strains, *egl-18(ga97)* and *egl-18(n162)*[55], we found that GFP expression in P5 - P8 in the embryo decreased from 94% for control animals to 60% in *egl-18(ga97)* and 68% in *egl-18(n162)* (Figure 5G and H, Table 3). These results suggest that EGL-18 is also required for proper *lin-39* expression in the embryo. To test for redundant function in *lin-39* regulation, we performed RNAi for one gene in the background of a mutant for the other (because the genes are adjacent, we were unable to build *egl-18*; *elt-6* double mutant animals). In neither case was there a significant decrease in the number of embryos showing *pJW3.9::GFP* expression compared to the *egl-18* or *elt-6* mutant strain treated with control vector (Table 3). Thus we did not obtain evidence for functional redundancy of *elt-6* and *egl-18* in regulating *pJW3.9::GFP* expression in the embryo, even though reduction of function for either gene alone affects embryonic GFP expression.

### ELT-6 and EGL-18 also regulate *lin-39* expression post-embryonically

We also examined whether *elt-6* affects *lin-39* expression post-embryonically. *elt-6* RNAi was performed on *lin-39::GFP* L1 larvae and we observed GFP expression in the VPCs in the same animals at the L3 stage. A statistically significant decrease in the number of VPCs (P5.p - P8.p) showing a wild type level of *lin-39::GFP* expression in the VPCs at the L3 stage was seen for *elt-6(L1 RNAi)* animals (Figures 2H and 3G), indicating that *elt-6* also positively regulates *lin-39* in the larval VPCs. EGL-18 is also likely to regulate *lin-39* expression postembryonically, since qRT-PCR on animals in which both *egl-18* and *elt-6* function is compromised had lower expression of *lin-39* than *elt-6*(*gk723*) mutant animals alone (Figure 4B).

To test the hypothesis that *lin-39* is a downstream target of ELT-6 and/or EGL-18 in the larva, we asked if overexpression of the GATA factors could increase *lin-39* levels. We overexpressed *egl-18* and *elt-6* using the heat shock promoter and assayed *lin-39* levels by qRT-PCR for *lin-39* one hour after a single heat shock at the L2/L3 molt. We found that *lin-39* levels went up 1.8 fold when ELT-6 was over-expressed, 1.6 fold when EGL-18 was over-expressed, and 1.7 fold when both ELT-6 and EGL-18 were over expressed (Figure 4C), supporting the hypothesis that *lin-39* is a downstream target of both ELT-6 and EGL-18 in the larval VPCs.

## Discussion

The *C. elegans* Hox gene *lin-39* functions in the midbody region of the developing *C. elegans* larva, where it is expressed in the P cells and their descendants, including the hermaphrodite vulval precursor cells (VPCs) [14]. During vulval development, *lin-39* expression is regulated by Wnt and Ras signaling pathways to facilitate VPC fate specification [26,28,45,46]. To further understand the function of *lin-39* in hermaphrodite vulval development, we wish to identify cis-acting sites and trans-acting factors required for initiation, maintenance and regulated expression of *lin-39* in the P cells and VPCs. Previously, using an enhancerless GFP reporter assay, we identified a *lin-39* enhancer fragment (JW5) that directs expression in the VPC P6.p at the time of vulval induction, which responds to Ras pathway activity, and which is bound by the Ras pathway effectors LIN-1 and LIN-31, as well as by LIN-39 itself [47]. We also identified a *lin-39* enhancer (JW3.9) that directs expression in the P cells in the embryo and in their larval descendants (the VPCs and ventral cord neurons), and showed that expression in the embryo depended on an evolutionarily conserved site that contains within it a putative GATA transcription factor binding site.

Here we took a complementary approach to identify additional factors that may regulate *lin-39* expression during vulval development. We concentrated on 27 short, evolutionarily conserved regions (ECRs) that we previously identified in the *lin-39* gene in *C. elegans*, *C. briggsae* and *C. remanei*[47], on the assumption that some may represent binding sites for phylogenetically conserved transcriptional regulators. We used the yeast one-hybrid technique to identify transcription factors that could bind to DNA fragments containing one or more of these ECRs, circumventing the requirement that the DNA site be sufficient to direct *in vivo* reporter expression [53]. Having found multiple transcription factors that interact with *lin-39* DNA fragments, we looked for effects on endogenous *lin-39* transcript levels and on expression from a *lin-39::GFP* reporter *in vivo* when the function of these proteins was reduced. Using this approach we identified six transcription factors that bind *lin-39* promoter sequences in yeast and *in vitro* and regulate *lin-39* expression and/or function *in vivo* (Figure 7A, Table 1). Three factors, the orphan nuclear receptor NHR-43, the hypodermal cell fate regulator LIN-26 and the GATA factor ELT-6 positively regulate *lin-39* expression in the embryo. In addition to those three factors, we identified the zinc finger proteins ZTF-17 and BED-3 and the T box factor TBX-9 as positive regulators of *lin-39* expression in the larval VPCs. Before this work, only four transcription factors were known to directly bind at the *lin-39* gene and regulate its expression; LIN-1, LIN-31, LIN-39 and TRA-1, all of which act during larval life (Figure 6B) [37,47]. Therefore, by this approach combining phylogenetic conservation and yeast one-hybrid screening, we have more than doubled the number of factors known to directly bind to and regulate *lin-39*, and for the first time we have identified factors that regulate expression of this Hox gene in the embryo.

We believe these factors regulate *lin-39 in vivo* because we observed a reproducible change in expression of the full length *lin-39::GFP* reporter and/or a decrease in endogenous *lin-39* transcript levels when the function of each of these factors was reduced, and in several cases we observed a phenotype in a LIN-39 regulated process (prevention of VPC fusion). Given their binding to sites from the *lin-39* genomic region, the simplest model would be that these factors directly regulate *lin-39* expression in the cells we examined (Figure 7A). However, in the current work we did not pursue *in vivo* binding studies for any of these factors; although published results from the modENCODE project show binding of ALR-1 to the evolutionarily conserved site we identified at the relevant time in development [40,63]. Additionally, while existing GFP reporters for four of these proteins (TBX-9, NHR-43, ALR-1 and ZTF-17) are turned on in some *lin-39*-expressing cells, expression in the P cells or VPCs has not been directly observed [57,59,61,64,67]. GFP reporters suffer from the caveat that all the elements required to recapitulate endogenous expression may not be present, or the expression may be weak or dynamic. However, until we can verify expression of each transcription factor in the embryonic P cells or larval VPCs, and show evidence of binding to the sites we identified *in vivo*, it remains possible that some of these factors regulate *lin-39* indirectly via another transcription regulator, or even act non cell-autonomously on *lin-39* expression. The strongest case for direct regulation can be made for BED-3, which regulates *lin-39* expression during larval life, and for LIN-26 and ELT-6, which regulate *lin-39* expression in the embryonic P cells.

### BED-3

*bed-3* encodes a 599 amino acid zinc finger protein with a BED DNA binding domain, and *bed-3* mutants show an Egl- laying (Egl) defective phenotype and defects in the terminal cell divisions of the descendants of the VPCs [68]. An intronic enhancer element from *bed-3* directs GFP expression in VPC descendants, leading to the hypothesis that BED-3 functions in the terminal cell divisions of vulval cells during vulval induction [68]. We found that *bed-3(RNAi)* showed the strongest effect on full length *lin-39::GFP* expression in the VPCs; in wild type animals the VPCs P5.p - P8.p showed lin-39::GFP expression 89% of the time (averaged over all four cells), while in *bed-3(RNAi)* animals these cells showed GFP expression only 37% of the time. This suggests that BED-3 could be acting at an earlier stage in vulval development. Consistent with this, we observed that the *bed-3 enhancer::GFP* reporter does show expression in the VPCs before they divide (Additional file 8: Figure S7), which would be consistent with BED-3 acting upstream of *lin-39* in the VPCs themselves. Therefore, although we could not verify BED-3 binding to the YF10 site *in vitro* with purified protein, we believe BED-3 is likely to be a positive regulator of *lin-39* in the larval VPCs before vulval induction, as well as functioning in the subsequent cell division of their progeny, as previously reported [68].

### LIN-26

LIN-26 is a 438 amino acid zinc finger transcription factor that shows continuous expression in all embryonic and larval hypodermal cells after their birth [65]. Loss of *lin-26* causes hypodermal cells to adopt incorrect cell fates or degenerate after their birth, resulting in embryonic lethality [65,66]. Ectopic expression of LIN-26 in the early embryo can induce cells to adopt hypodermal-like fates [72]. These results suggest that LIN-26 is required to specify and/or maintain the hypodermal cell fate [65]. *lin-26* itself is positively regulated by the GATA factor ELT-1, which is another global regulator of hypodermal cell fate [73]. We found that *lin-26* RNAi caused a weak but significant decrease in the expression of the full length *lin-39::GFP* reporter in the P cells in the embryo; 100% of wild type embryos show GFP expression, compared to 86% of *lin-26(RNAi)* animals. LIN-26 binds *in vitro* to a 51 bp sequence located approximately 2 kb upstream of the *lin-39* start codon (Figure 6B). Recent data recording reporter gene expression from live developing embryos shows that a *lin-26::mCherry* transcriptional reporter shows expression in the mothers of the P cells shortly before they divide, at the same time as these cells also begin to show expression of a *lin-39*:*:mCherry* reporter [74] (Additional file 9: Figure S8). Based on these results, and the known function of LIN-26 in hypodermal cell fate, we propose that LIN-26 positively regulates *lin-39* expression in the embryonic P cells, and that the function of this regulation is to aid in initial P cell fate specification and/or to maintain the P cell identity once established.

### ELT-6

*elt-6* encodes a 367 amino acid GATA family transcription factor. The *elt-6* gene is immediately downstream from another GATA factor gene, *egl-18* (Figure 7B), and reporter gene experiments suggest *elt-6* may be expressed on its own and as part of a dicistronic message with *egl-18*[70]. These genes are expressed in many cells in the embryo, including the descendants of the MS and AB blastomeres (which give rise to the P cells). In the larva, they are expressed strongly in the lateral hypodermal seam cells and weakly in the VPCs [55,70]. These two GATA factors share 76% identity in their DNA-binding domains, and have been shown to act redundantly in seam cell development in both the embryo and larva [70,71]. We previously showed that a 340 bp *lin-39* enhancer (JW3.9) directs GFP expression in the embryonic P cells, and a conserved GATA site in the enhancer was necessary for expression [47]. Here we identify the GATA factor ELT-6 as binding to this *lin-39* enhancer in yeast and *in vitro*, and show that binding was dependent on the GATA sequence. *elt-6* RNAi showed a decrease in expression of the full length *lin-39::GFP* reporter in embryonic P cells, and this result was recapitulated with the *pJW3.9* reporter in *elt-6*(*gk723*) mutant and *elt-6(RNAi)* embryos. These data suggest that ELT-6 is required for proper expression of the Hox gene *lin-39* in the embryonic P cells. Consistent with this, recent data from live recordings of developing embryos shows that *elt-6* expression in the mothers of P3 - P8 begins before *lin-39* expression is first seen ([74]; Additional file 8: Figure S7). This is the first example to our knowledge of a phenotype caused by reduction of *elt-6* function alone. *elt-6* RNAi treatment of newly hatched L1 larvae also led to a weak reduction in *lin-39::GFP* expression in the L3 VPCs, and overexpression of ELT-6 or EGL-18 increased endogenous *lin-39* expression in larvae. These results suggest that ELT-6 and/or EGL-18 may continue to positively regulate *lin-39* expression in larval life in the VPCs.

Given these results, it is interesting that we previously showed, in collaboration with the Rothmann laboratory, that the *egl-18*/*elt-6* locus was likely to be a downstream target of LIN-39 in the larval VPCs [55]. In that work an 800 bp enhancer element was identified in intron 2 of the *egl-18* gene that directs GFP expression in the VPCs and their descendants starting in the L2 stage. This enhancer contains two Hox protein-binding sites that are bound *in vitro* by LIN-39 and its binding partner CEH-20, and mutation of one site eliminated enhancer-driven GFP expression. Finally, overexpression of *egl-18* from the heat shock promoter was able to partially rescue vulval defects in *lin-39(RNAi)* animals. These data led to the model that *egl-18*/*elt-6* is a downstream target of LIN-39 during vulval development.

Combining these previous data with our current results, our working model suggests LIN-26 and ELT-6 are involved in the initiation of expression of the Hox gene *lin-39* in the P cells in the embryo (Figure 7A). *lin-39* may also be regulated by EGL-18 at this time, since *egl-18* mutants had reduced enhancer-driven GFP expression in the P cells, and EGL-18 protein bound to the *pJW3.9* GATA site *in vitro* (W. Liu and D. Eisenmann, unpublished results). Once the fate of these cells is established, a positive feedback loop is established between ELT-6, acting via the upstream *lin-39* enhancer *pJW3.9*, and LIN-39, acting via the intronic enhancer in *egl-18*[55]. We propose that this positive feedback loop helps maintain expression of these genes and the fate of these cells and their descendants, the VPCs, during subsequent embryonic and larval development (Figure 7B). Interestingly, we have seen another example of *lin-39* feedback regulation. The zinc finger protein SEM-4 was previously shown to positively regulate *lin-39* expression in VPCs [49]. We have found that in animals overexpressing either LIN-39 or LIN-39 and CEH-20, *sem-4* expression is decreased, suggesting that feedback mechanisms to decrease *lin-39* levels when they are elevated may also exist (J. Siegel and D. Eisenmann, unpublished results). Recent chromatin immunoprecipitation experiments from larvae verify binding of tagged LIN-39 protein upstream and in introns of both the *egl-18* and *sem-4* genes [40,63].

## Conclusion

While much is known about the initiation and regulation of Hox gene expression in Drosophila and vertebrates, less is known outside of these well-studied groups. Our laboratory and others have been studying the expression and function of the Hox gene *lin-39* during vulval development in the nematode *C. elegans*. We used the yeast one-hybrid (Y1H) method to identify 16 transcription factors that interact with specific regions of the *lin-39* gene, and further characterized several factors (ALR-1, BED-3, ELT-6, LIN-26, NHR-43, TBX-9 and ZTF-17) showing that their function is required for proper expression of *lin-39::GFP* reporters and endogenous *lin-39 in vivo*. This work greatly expands the number of factors known to directly regulate *lin-39* expression. Given the known caveats of the yeast one-hybrid technique (absence of specific posttranslational modifications, lack of heterodimeric binding factors), and our emphasis on characterization of factors with known expression or phenotypes in the vulval cells, it is likely that there are additional factors that regulate *lin-39* in the embryo and larva that we did not identify in our screens. Given the important role of Hox genes in patterning the developing metazoan body, it is not surprising that Hox gene expression is found to be complicated in those species where it has been examined closely. Our results suggest that expression of the *C. elegans* Hox gene *lin-39* in the P cells and VPCs during vulval development may be regulated by a large number of transcription factors, each making a small contribution to overall *lin-39* expression on its own. This is consistent with *in vivo* data from *C. elegans* showing that the average worm gene is bound by several transcription factors at one time in larval development [40,63]. Such a mechanism may ensure a robustness of expression for important developmental regulators like Hox genes. Transcription factors behaving in this manner would also not be identified in genetic screens, since each makes a small overall contribution to *lin-39* expression, and feedback mechanisms may exist to compensate for reductions in *lin-39* transcript levels. Finally, for the first time, we have identified factors required for *lin-39* expression in the embryo (NHR-43, LIN-26 and ELT-6), and our results with EGL-18 and ELT-6, combined with our earlier work on LIN-39 regulation of *egl-18*/*elt-6*, hint at a positive feedback mechanism to maintain *lin-39* expression in the vulval lineages. The identification of LIN-26, ELT-6, and NHR-43 will help us further characterize the mechanisms for the initiation of Hox gene expression in the nematode, allowing us to make comparisons across metazoan phyla about the mechanisms utilized to regulate this essential class of developmental regulators.

## Methods

### Yeast one-hybrid (Y1H) assays

Y1H assays were performed using two methodologies: a traditional library ‘transformation’ screen, and a robotically-assisted ‘mating’ screen (“enhanced” Y1H, eY1H). Briefly, each DNA fragment of interest (YF1 - YF12, *pJW3.9*; see Additional file 1: Figure S1) was cloned into reporter vectors pMW#2 (*HIS3*) and pMW#3 (*LacZ*) and the resulting constructs were sequentially integrated into the genome of yeast strains BY5444 and YM4271 to generate thirteen “DNA bait” strains. BY5444 and YM4271 are isogenic for multiple marker genes (*MATa ura3-52 his3-200 ade2-101 lys2-801 leu2-3,112 trp1-901 tyr1-501 gal4-Δ512 gal80-Δ538 ade5::hisG*) but BY5444 does not mate efficiently with the Yalpha1867 strain used in eY1H assays.

The ‘transformation’ screen was performed as described [53]. Each of the thirteen bait strains (BY5444 background) was transformed with DNA from a commercially-available, *C. elegans* transcription factor library containing 755 plasmids that each express one *C. elegans* transcription factor fused in frame with the yeast GAL4 activation domain (Open Biosystems; [75]). Potential interactions were identified as those colonies that turned blue on plates containing X-Gal, and which grew on plates containing a higher concentration of 3-aminotriazole than the control strain grew on. The eY1H screens were performed as described [54]. Each of the thirteen bait strains (both BY5444 and YM4271 backgrounds) was mated with a collection of 936 Y1Halpha1867 strains of the opposite mating type, each of which expresses a single *C. elegans* transcription factor fused to the GAL4 activation domain. Matings were done in quadruplicate. Potential interactions were identified as those for which at least two of the four colonies exhibited higher expression of both reporters than control yeast, as assayed by blue color and growth on SC-His + 5 mM 3-aminotriazole + X-Gal. For both methods, plasmids were recovered from positive yeast, sequenced to identify the *C. elegans* gene, and then retransformed back into the appropriate yeast strain to confirm the interaction. From both screens, only those interactions that repeated after retransformation were considered true positives. For the haploid transformation screen, while most baits gave many 3-aminotriazole resistant colonies, only RF1, RF2, RF4 and RF6 gave double positive colonies, and only interactions with NHR-43, ALR-1 and ZTF-17 repeated (all three factors were identified multiple times). For the robotically assisted mating screens, the results are presented in Additional file 4: Table S2-S4.

### Genetic methods, alleles and strains

Methods for culture and genetic manipulation of *C. elegans* were performed as described [76]. Bristol variety (strain N2) of *C. elegans* was used as wild-type. Experiments were performed at 20°C unless noted. Genes and alleles used in this work are described in [77] and Wormbase [57].

LGII*: rrf-3(pk1426), lin-26(n56)*

LGIII: *tbx-9(ok2473), tbx-9(ms31), unc-119(ed3), pha-1(e2123), lin-39(n709ts)*

LGIV: *nhr-43(tm1381), bed-3(sy702), bed-3(sy705), elt-6(gk723), egl-18(ga97), egl-18(n162), egl-18(ok290)*

LGX: *alr-1(ok545)*

Strains used:

*lin-39::GFP*: *smg-1(e1228); him-5(e1490); deIs4[lin-39TN::GFP; dpy-20(+); ajm-1::GFP]*[46]

*nhr-43::gfp: unc-119(ed3); Ex[C29E6.5::gfp; unc-119 (+)]*[64]

*bed-3::GFP: unc-119(ed3); syEx962[pTI06.29; unc-119(+); pBSKSII(+)]*[68]

*pJW3.9::GFP*: *pha-1(e2123); deEx105[pJW3.9::gfp; pha-1(+); ajm-1::GFP]*[47]

Strains created for this work:

hs::control: unc-119(ed3); deEx106[pPD49.78; unc-119(+); ajm-1::GFP]

hs::elt-6: unc-119(ed3); deEx107[pLG1; unc-119(+), ajm-1::GFP]

hs::egl-18: unc-119(ed3); deEx108[pPK8; pPK9; unc-119(+); ajm-1::GFP]

hs::egl-18+ hs::elt-6: unc-119(ed3); deEx109 [pLG01; pKK8; pKK9 unc-119 (+); ajm-1::GFP]

### RNA interference

RNA interference *(RNAi)* was performed using the bacterial ‘feeding’ method in which dsRNA for the gene of interest is produced in *E. coli* strain HT115 and ingested by worms [78]. For feeding of L1 larvae, eggs from strains to be tested were placed on plates without *E. coli* for at least 18 hours at 20°C, then semi-synchronized L1 larvae were washed off, placed onto plates with the desired HT115 RNAi strain and grown at 20°C to the appropriate stage for scoring. For feeding of P0 animals, L1 stage P0 larvae were put onto the desired RNAi plates, grown to adulthood and their F1 progeny were analyzed at the appropriate stage.

### Reporter gene analysis

Worms carrying GFP reporter constructs were analyzed using fluorescence microscopy on a Zeiss Axioplan 2 at the desired developmental time. GFP expression in live animals was captured using a Nikon DXM 1200 digital camera and the ACT-1 program (version 2.12). For *deIs4* [*lin-39::GFP*] and *pJW3.9::GFP* reporter analysis, the percentage of animals showing an intensity of expression similar to wild-type was determined. For embryonic expression in the cells P5 - P8 the number of embryos showing wild type expression in all four cells was recorded. For larval expression in the cells P5.p - P8.p the intensity of GFP expression in the individual cells at the L3 stage was analyzed using ImageJ [79] and pixel counts for each cell were recorded (after subtraction of background). For each RNAi treatment, at least 20 animals at the L3 stage were photographed, under identical conditions. Expression data was statistically analyzed using an unpaired *t*-test and both P values and SD were gathered. All *deIs4* experiments were carried out in a *smg-1* mutant background, in which nonsense-mediated decay is abrogated, leading to more robust reporter expression [46].

### Creation of strains expressing EGL-18 and ELT-6 from the heat shock promoter

For heat-shock induced expression of EGL-18, we used the previously described constructs pKK8 and pKK9 in which the entire coding region of *egl-18* is inserted into the heat shock promoter vectors pPD49.78 and pPD49.83 respectively [55]. A similar heat shock expression construct, pLG1 (gift of L. Gorrepati), was made by cloning *elt-6* genomic DNA from the start to stop codon into vector pPD49.78 (available at [80]; gift of A. Fire, Stanford University School of Medicine, Stanford, CA). To create transgenic strains, DNA for these constructs was co-injected at 25 ng/ul with *unc-119 (+)* (100 ng/ul) and *ajm-1::gfp* DNA (50 ng/ul) [81] into *unc-119(ed3)* hermaphrodites. For the *hs::control* strain, the empty heat-shock vector pPD49.78 was used. For the *hs::elt-6 + hs::egl-18* strain, pLG1, pKK8 and pKK9 were injected together. Several lines rescued for the Unc phenotype were recovered for each injection and the ones with the highest percentage transmission to progeny were analysed.

### Heat shock protocol

Embryos from transgenic animals containing the arrays *hs::control, hs::elt-6, hs::egl-18,* and *hs::elt-6 + hs::egl-18* were collected from hypochlorite-treated gravid animals and hatched on NGM plates without food for at least 18 hours at 20°C, allowing for early L1 stage arrest. Synchronized L1 animals were fed with OP50, grown to the early L3 stage (25 hours post feeding at 20°C), heat shocked at 33°C for 30 minutes, then transferred back to 20°C and collected 30 minutes later for RNA isolation.

### RNA isolation

For genes with existing mutant strains, animals were synchronized and grown to early L3 stage (26 hours after feeding at 20°C) to be collected. For genes without existing mutant strains, RNAi was performed on wild type worms. For heat shocked worms, animals were treated with the heat shock program described above. Three biological replicates were performed for each strain and RNA was extracted using the RNAeasy mini kit (Qiagen).

### qRT-PCR

The mRNA fraction of extracted total RNA pools was reverse transcribed to cDNA using iScript™ cDNA synthesis kit (Bio-Rad) and used in triplicate qRT-PCR reactions run on an iCycleriQ real-time PCR machine (BioRad). Relative expression ratios were calculated from observed Ct values using the ΔΔCt method [fold change = (E_target_) ^targetΔCt (control-sample)^/ (E_ref_) ^RefΔCt (control-sample)^[82]] with the house keeping gene *gpd-2* as a reference [83]. For mutant strains, N2 animals were the control. For RNAi-treated worms, N2 animals treated with the empty RNAi ‘feeding vector’ (L4440) (available at [80]; gift of A. Fire, Stanford University School of Medicine, Stanford, CA) were the control. For heat shocked strains, *hs::control* animals which underwent the heat shock treatment were the control. Three biological replicates were used for each sample. The data was statistically analyzed using unpaired *t*-test and both P values and SD were gathered with Graphpad software. Primers for *gpd-2* and *lin-39* were *gpd-2*FW (CCTCTGGAGCCGACTATGTC), *gpd-2*RV (TGGCATGATCGTACTTCTCG), *lin-39*FW (CGGAGATCAGTCACTATGCT) and *lin-39*RV (CCGCGTGAACCTCCTGTAGT).

### Site direct mutagenesis

Site directed mutagenesis was performed using the Quick Change site directed technique following manufacturer’s instructions with the high fidelity DNA polymerase Pfu Turbo (Stratagene). Plasmid DNA was extracted and sequenced to confirm the presence of mutations.

### Protein purification

Full length cDNAs for *nhr-43*, *alr-1*, *ztf-17*, *lin-26*, *bed-3*, *tbx-9*, and *elt-6* were obtained by PCR amplification from plasmids obtained from the Open Biosystems Y1H library [75] and cloned individually into the plasmid pQE-80 L (Qiagen), which introduces a 6His tag at the N terminus. Plasmids were transformed into *E. coli* strain BL21, grown to an OD of 0.7 and induced with 1 mM IPTG for 4 hours. For purification of NHR-43, ALR-1, LIN-26, BED-3, TBX-9, and ELT-6, cells were centrifuged, resuspended in lysis buffer (50 mM, NaH_2_PO_4_, 300 mM NaCl, 10 mM imidazole, pH 8.0 plus protease inhibitors [Sigma]), sonicated for 3 minutes on a Branson Sonifier 450, and centrifuged. The supernatant were collected and loaded on a Ni^2+^-NTA agarose column (Qiagen), washed five times with wash buffer (50 mM NaH_2_PO_4_, 300 mM NaCl, 20 mM imidazole, pH 8.0) and eluted with elution buffer (50 mM NaH_2_PO_4_, 300 mM NaCl, 250 mM imidazole, pH 8.0). For purification of ZTF-17, cells were centrifuged, resuspended in sonication buffer (20 mM Tris 7.9, 500 mM NaCl), sonicated and centrifuged. The pellet was resuspended in solubilizing buffer (6 M Guanidine, 20 mM Tris 7.9, 300 mM NaCl, 5 mM Imidazole, 5% glycerol plus protease inhibitors [Sigma]). Insoluble material was pelleted by centrifugation and the supernatant was loaded onto a Ni^2+^-NTA agarose column, washed five times with buffer and eluted with elution buffer 2(6 M Guanidine, 300 mM NaCl, 300 mM Imidazole, 40% glycerol, 20 mM HEPES pH7.5). Protein was diluted to 0.5 mg/ml with elution buffer, dialyzed at 4°C overnight against dialysis buffer (20 mM HEPES pH7.5, 300 mM NaCl, 40% glycerol) and concentrated to 500 ng/ul using a Centricon-10.

The primers for cDNA amplification were:

NHR-43: FW:GCGCGGATCCATTAGCGGCCCATTTCTTCAC/RV: GCGCGCAAGCTTTTAGATTGAGTACAAGTAGGC

ALR-1: FW: GCGCGCATGCCCCGAGTTGAAGAAAGAAGA/RV: GCGCGCAAGCTTTCATGAACTTTCTTCTTTTG

ZTF-17: FW: GCGCGCATGCCTGCGCTACCAGGCGTCCGTG/RV:CGCCCCGGGCTATTTTACTCTAAGAAATA

LIN-26: FW: GCGCGCATGCCTTTCTAAATTTGTGGTAGTC/RV: CGCCCCGGGCTACACCAATGGTTGAGCCAT

TBX-9: FW: GCGCGAGCTCTCCAAAGTCAAAGTATCA/RV: CGCCCCGGGTCAACCAACAATATCAAT

BED-3: FW: GCGCGCATGCCAGACCCAAAGTCCATTT/RV: CGCCCCGGGTCAAACAAGTTGATCAAT

ELT-6: FW: GCGCGCATGCACGTCGTCGAAGGAGGAGATG/RV: CGCCCCGGGTCAGGGAGACTTGCGCTGCTC

### Electrophoretic mobility shift assays

For DNA probes larger than 100 bp, DNA fragments were produced by PCR, and 5 pmole was labeled with ^32^P using T4 polynucleotide kinase (NEB). For DNA probes less than 100 bp, one oligonucleotide was labeled with ^32^P, then annealed with the complementary oligonucleotide. The labeled double stranded DNA probes were purified using Centri-Spin-20 columns (Princeton Separation), and their activity was measured using a standard scintillation counter. The oligonucleotides used to make EMSA probes are listed in Additional file 5: Figure S6.

DNA binding reaction were set up at 4°C in a volume of 20 ul in final buffer conditions of 50 mM KCl, 20 mM HEPES, pH 7.9, 0.2 mM EDTA, 0.5 mM DTT, 3 mM MgCl_2_, 1 μg poly(dI–dC), 0.5 μg/ul BSA, 5000 cpm ^32^P-labeled DNA probe and 0–800 ng of protein. Reactions were incubated 20 minutes on ice, then loaded onto 4.5% (probe size smaller than 100 bp) or 6% (probe size larger than 100 bp) polyacrylamide gels. Gels were run in 0.5xTBE buffer at 150 V for 2.5 hours (4.5% gel), or 220 V for 2 hours (6% gel), dried and analyzed using a Storm 860 phosphorimager (Molecular Dynamics). For competitive binding experiments, the indicated concentration of cold competitor was added to the reaction before the protein.

### VPC fusion assay in lin-39 sensitized background

For *nhr-43*, *alr-1*, *ztf-17*, *tbx-9*, *elt-6* and control RNAi, P0 *lin-39(n709ts)*; *wIs79(ajm-1::GFP)* larvae were grown to adulthood at 25°C on the desired RNAi plates, then semi-synchronized F1 progeny were grown to the late L2 stage, when *ajm-1::GFP* expression in the vulval precursor cells was examined (17 hours post feeding). For *bed-3* and *lin-26* RNAi, *lin-39(n709ts)*; *wIs79(ajm-1::GFP)* eggs were hatched in M9 overnight at 20°C, then semi-synchronized L1 larvae were washed off, placed onto plates with the desired HT115 RNAi strain and grown at 25°C to the late L2 stage and scored. At least 25 animals were scored for each RNAi treatment using fluorescence microscopy on a Zeiss Axioplan 2. Wild type animals have either 5 or 6 VPCS with *ajm-1::GFP* expression at this time (due to fusion of P3.p with hyp7). The percentage of animals lacking *ajm-1:: GFP* expression in P4.p - P8.p was determined. The data was statistically analyzed using Fisher’s exact test. 2X2 contingency tables were analyzed using Fisher’s exact test and P values were gathered.

## Competing interests

The authors declare that they have no competing financial or non-financial interests.

## Authors’ contributions

JRH carried out all robotically-assisted yeast one hybrid screens. WJL performed all other work. WJL and DME wrote the manuscript. All authors read and approved the final manuscript.

## Supplementary Material

Additional file 1: Figure S1 *lin-39* genomic region and fragments used in yeast one-hybrid screens. The top line shows 26 kb around the *lin-39* locus with base locations on chromosome II shown. Two *lin-39* transcripts and the upstream microRNA gene *mir-231* are diagrammed below. JW1-10 (bottom) are *lin-39* genomic regions previously used in reporter gene analysis*;* those in green drove GFP expression *in vivo* in *lin-39* expressing cells [47]. *pJW3.9* (orange box) is a 340 bp subfragment that drove GFP expression in P5 – P8 in the embryo [47]. Thirty-one evolutionarily-conserved regions (ECRs) with >75% identity in the *lin-39* gene from three *Caenorhabditis* species were previously identified [47]. ECRs are shown on the middle line as unlabeled, vertical black lines. Twenty-seven ECRS were grouped into 12 PCR fragments (numbered 1–12; red boxes). The twelve fragments (YF1-12) and *pJW3.9* were used as ‘baits’ in Y1H screens. Click here for file

Additional file 2: Figure S2 Fourteen transcription factors interact with *lin-39* genomic fragments in yeast one-hybrid assays (strain BY5444). Thirteen strains in background BY5444 containing fragments (YF1 - 12, *pJW3.9*) from the *lin-39* genomic region were screened for interactions with *C. elegans* transcription factors by both the haploid library transformation method, and the robotically-assisted mating method (see Methods). Any positive interactions from primary screens were retested by retransformation of the rescued interacting plasmid back into the appropriate ‘bait’ strain (see Additional file 4: Table S2). Shown here are three-fold serial dilutions of each strain grown on SC–His-Ura-Trp plates with no 3AT (control plates), SC–His-Ura-Trp with 10 mM 3AT, and plates with X-gal. Bait strains transformed with the pDEST-AD empty vector were the ‘Control’. Positive interactions were considered those showing more growth on 3AT and/or more blue color on XGal than the control. Note that the YF8 strain shows considerable self-activation on 3AT, but positive interactions for NHR-111 and FLH-1 are observable on the XGal plates. Click here for file

Additional file 3: Figure S3 Four transcription factors interact with *lin-39* genomic fragments in yeast one-hybrid assays (strain YM4271). Thirteen strains in background YM4271 containing fragments (YF1 - 12, *pJW3.9*) from the *lin-39* genomic region were screened for interactions with *C. elegans* transcription factors by the robotically-assisted mating method (see Methods). Positive interactions from the primary screen were retested by retransformation of the rescued interacting plasmid back into the appropriate ‘bait’ strain (see Additional file 4: Table S3). Shown here are three-fold serial dilutions of each strain grown on SC–His-Ura-Trp plates with no 3AT (control plates), SC–His-Ura-Trp with 10 mM 3AT, and plates with X-gal. Bait strains transformed with the pDEST-AD empty vector were the ‘Control’. Positive interactions were considered those showing more growth on 3AT and/or more blue color on XGal than the control. Click here for file

Additional file 4: Table S1 Sequences of 12 YFs and *pJW3.9*. **Table S2.** Yeast one-hybrid screen with strain BY5444 (screen YM2). **Table S3.** Yeast one-hybrid screen with strain YM4271 (screen YM2). **Table S4.** Summary of yeast one-hybrid screen results. **Table S5.** Only GATA factor ELT-6 binds *pJW3.9* in yeast*. elegans* GATA factors. **Table S6.** Oligonucleotides for EMSA probes. Click here for file

Additional file 5: Figure S6 TBX-9 binds a 79 bp region in fragment YF9. A) The top line shows fragment YF9 and the location of ECRs 21 – 23. Subfragments A – D are diagrammed below. Shading indicates the fragment bound by TBX-9 *in vitro*; B) Gel mobility shift assay with TBX-9 protein purified from *E. coli* and labeled fragment YF9 (lane 2) and competition with subfragments A – D (lanes 3–6)*.* Arrowhead indicates free probe; arrow indicates the protein DNA complex that can be competed by fragment C. Two other bands appear with added TBX-9 protein: the upper band is competed away by all four competing fragments, while the lower band is competed by none of the fragments. The nature of these complexes is unknown, although they are likely to represent non-specific binding by TBX-9 or another protein. C) Gel mobility shift assay with TBX-9 protein and labeled fragment subfragments A – D*.* The bracket indicates the migration locations of free probes (which differ in size, see panel A). Arrow indicates a complex with TBX-9 and subfragment C. Click here for file

Additional file 6: Figure S4 ZTF-17 binds to fragment YF4-4 in yeast. BY5444 ‘bait’ strains containing subfragments of YF1 (YF4-1 to YF4-4; see Figure 1) were made and transformed with the plasmid encoding ZTF-17::Gal4AD or with the pDEST-AD empty vector as control. Three-fold serial dilutions of each strain grown on SC–His-Ura-Trp plates with no 3AT (control plates), SC–His-Ura-Trp with 10 mM 3AT, and plates with X-gal are shown. Fragment YF4-1 shows strong self-activation on 3AT. Only fragment YF4-4 shows a strong interaction with ZTF-17 on both 3AT and XGal plates. Click here for file

Additional file 7: Figure S5 LIN-26 binds to fragment YF4-3 using yeast. BY5444 ‘bait’ strains containing subfragments of YF1 (YF4-1 to YF4-4; see Figure 1) were made and transformed with the plasmid encoding LIN-26::Gal4AD or with the pDEST-AD empty vector as control. Three-fold serial dilutions of each strain grown on SC–His-Ura-Trp plates with no 3AT (control plates), SC–His-Ura-Trp with 10 mM 3AT, and plates with X-gal are shown. Fragment YF4-1 shows strong self-activation on 3AT. Only fragment YF4-3 shows a strong interaction with LIN-26 on both 3AT and XGal plates. Click here for file

Additional file 8: Figure S7 *bed-3::GFP* expression in the larval VPCs. Expression from strains carrying *syEx962* which contains the *bed-3::GFP* reporter pTI06.29 [68]. GFP expression is seen in the Pn.p cells in the L1 stage (A) and L2 stage (B), including the VPCs P3.p - P8.p (indicated by white bars). Click here for file

Additional file 9: Figure S8 Expression of transcription factor genes *elt-1, elt-3, lin-26* and *lin-39* in the ABpra lineage in developing embryos. The images shown are taken directly from the Expression Patterns in Caenorhabditis web site [84]. As described [74], live images were recorded from developing embryos that expressed a histone:mCherry fusion protein driven from the upstream promoter sequences for each of the indicated genes. Expression levels in individual embryonic cells were characterized and diagrammed on the cell lineage chart. An expression scale for all experiments is shown at the top and shows the fluorescence intensity of the reporter construct in the individual cells; time (in minutes) for each experiment is shown along the left. The data shown are from the following experiments: 20080128_elt-1_3.html, 20070817_*elt-6*_5.html, 20080805_*lin-26*_5_L1.html and 20071015_*lin-39*_9. Click here for file

## References

[B1] Hueber SD Lohmann I Shaping segments: Hox gene function in the genomic age Bioessays 2008 30 10 965 979 10.1002/bies.20823 18798525

[B2] McGinnis W Krumlauf R Homeobox genes and axial patterning Cell 1992 68 2 283 302 10.1016/0092-8674(92)90471-N 1346368

[B3] Foronda D de Navas LF Garaulet DL Sanchez-Herrero E Function and specificity of Hox genes Int J Dev Biol 2009 53 8–10 1404 1419 19247930 10.1387/ijdb.072462df

[B4] Pearson JC Lemons D McGinnis W Modulating Hox gene functions during animal body patterning Nat Rev Genet 2005 6 12 893 904 10.1038/nrg1726 16341070

[B5] Deutsch J Deutsch J Hox Genes: Studies from the 20th to the 21st Century Advance in Experimental Medicine and Biology, Volume 689 2010 Austin, Texas USA: Landes Bioscience 20795318

[B6] Gellon G McGinnis W Shaping animal body plans in development and evolution by modulation of Hox expression patterns Bioessays 1998 20 2 116 125 10.1002/(SICI)1521-1878(199802)20:2<116::AID-BIES4>3.0.CO;2-R 9631657

[B7] Kmita M Duboule D Organizing axes in time and space; 25 years of colinear tinkering Science 2003 301 5631 331 333 10.1126/science.1085753 12869751

[B8] Tschopp P Duboule D A genetic approach to the transcriptional regulation of Hox gene clusters Annu Rev Genet 2011 45 145 166 10.1146/annurev-genet-102209-163429 22060042

[B9] Akam M The molecular basis for metameric pattern in the Drosophila embryo Development 1987 101 1 1 22 2896587

[B10] Deschamps J van den Akker E Forlani S De Graaff W Oosterveen T Roelen B Roelfsema J Initiation, establishment and maintenance of Hox gene expression patterns in the mouse Int J Dev Biol 1999 43 7 635 650 10668974

[B11] Brock HW Fisher CL Maintenance of gene expression patterns Dev Dyn 2005 232 3 633 655 10.1002/dvdy.20298 15704101

[B12] Schuettengruber B Chourrout D Vervoort M Leblanc B Cavalli G Genome regulation by polycomb and trithorax proteins Cell 2007 128 4 735 745 10.1016/j.cell.2007.02.009 17320510

[B13] Carroll S Grenier JK Weatherbee SD From DNA to Diversity: Molecular Genetics and the Evolution of Animal Design 2001 Malden, MA: Blackwell Science

[B14] Kenyon CJ Austin J Costa M Cowing DW Harris JM Honigberg L Hunter CP Maloof JN Muller-Immerglück MM Salser SJ Waring DA Wang BB Wrischnik LA The dance of the Hox genes: patterning the anteroposterior body axis of *Caenorhabditis elegans* Cold Spring Harb Symp Quant Biol 1997 62 293 305 9598363

[B15] Aboobaker A Blaxter M Hox gene evolution in nematodes: novelty conserved Curr Opin Genet Dev 2003 13 6 593 598 10.1016/j.gde.2003.10.009 14638320

[B16] Aboobaker AA Blaxter ML Hox Gene Loss during Dynamic Evolution of the Nematode Cluster Curr Biol 2003 13 1 37 40 10.1016/S0960-9822(02)01399-4 12526742

[B17] Brunschwig K Wittmann C Schnabel R Bürglin T Tobler H Müller F Anterior organization of the *Caenorhabditis elegans* embryo by the labial-like Hox gene ceh-13 Development 1999 126 7 1537 1546 10068646 10.1242/dev.126.7.1537

[B18] Van Auken K Weaver DC Edgar LG Wood WB *Caenorhabditis elegans* embryonic axial patterning requires two recently discovered posterior-group Hox genes Proc Natl Acad Sci U S A 2000 97 9 4499 4503 10.1073/pnas.97.9.4499 10781051 PMC18263

[B19] Wittmann C Bossinger O Goldstein B Fleischmann M Kohler R Brunschwig K Tobler H Müller F The expression of the *C. elegans* labial-like Hox gene *ceh-13* during early embryogenesis relies on cell fate and on anteroposterior cell polarity Development 1997 124 21 4193 4200 9334268 10.1242/dev.124.21.4193

[B20] Clark SG Chisholm AD Horvitz HR Control of cell fates in the central body region of *C. elegans* by the homeobox gene *lin-39* Cell 1993 74 1 43 55 10.1016/0092-8674(93)90293-Y 8101475

[B21] Ferreira H Zhang Y Zhao C Emmons S Patterning of *Caenorhabditis elegans* posterior structures by the Abdominal-B homolog, *egl-5* Dev Biol 1999 207 1 215 228 10.1006/dbio.1998.9124 10049576

[B22] Wang BB Muller-Immergluck MM Austin J Robinson NT Chisholm A Kenyon C A homeotic gene cluster patterns the anteroposterior body axis of *C. elegans* Cell 1993 74 1 29 42 10.1016/0092-8674(93)90292-X 8101474

[B23] Streit A Kohler R Marty T Belfiore M Takacs-Vellai K Vigano M Schnabel R Affolter M Müller F Conserved regulation of the *Caenorhabditis elegans* labial/Hox1 gene ceh-13 Dev Biol 2002 242 2 96 108 10.1006/dbio.2001.0544 11820809

[B24] Salser S Loer C Kenyon C Multiple HOM-C gene interactions specify cell fates in the nematode central nervous system Genes Dev 1993 7 9 1714 1724 10.1101/gad.7.9.1714 8103754

[B25] Ch’ng Q Kenyon C *egl-27* generates anteroposterior patterns of cell fusion in *C. elegans* by regulating Hox gene expression and Hox protein function Development 1999 126 15 3303 3312 10393110 10.1242/dev.126.15.3303

[B26] Eisenmann DM Maloof JN Simske JS Kenyon C Kim SK The beta-catenin homolog BAR-1 and LET-60 Ras coordinately regulate the Hox gene *lin-39* during *Caenorhabditis elegans* vulval development Development 1998 125 18 3667 3680 9716532 10.1242/dev.125.18.3667

[B27] Jiang LI Sternberg PW Interactions of EGF, Wnt and HOM-C genes specify the P12 neuroectoblast fate in *C. elegans* Development 1998 125 12 2337 2347 9584132 10.1242/dev.125.12.2337

[B28] Maloof JN Kenyon C The Hox gene *lin-39* is required during *C. elegans* vulval induction to select the outcome of Ras signaling Development 1998 125 2 181 190 9486792 10.1242/dev.125.2.181

[B29] Maloof JN Whangbo J Harris JM Jongeward GD Kenyon C A Wnt signaling pathway controls hox gene expression and neuroblast migration in *C. elegans* Development 1999 126 1 37 49 9834184 10.1242/dev.126.1.37

[B30] Alper S Kenyon C REF-1, a protein with two bHLH domains, alters the pattern of cell fusion in *C. elegans* by regulating Hox protein activity Development 2001 128 10 1793 1804 11311160 10.1242/dev.128.10.1793

[B31] Chamberlin HM Thomas JH The bromodomain protein LIN-49 and trithorax-related protein LIN-59 affect development and gene expression in *Caenorhabditis elegans* Development 2000 127 4 713 723 10648230 10.1242/dev.127.4.713

[B32] Chen Z Han M *C. elegans* Rb, NuRD, and Ras regulate *lin-39*-mediated cell fusion during vulval fate specification Curr Biol 2001 11 23 1874 1879 10.1016/S0960-9822(01)00596-6 11728311

[B33] Ross JM Zarkower D Polycomb group regulation of Hox gene expression in *C. elegans* Dev Cell 2003 4 6 891 901 10.1016/S1534-5807(03)00135-7 12791273

[B34] Zhang H Emmons SW The novel *C. elegans* gene *sop-3* modulates Wnt signaling to regulate Hox gene expression Development 2001 128 5 767 777 11171401 10.1242/dev.128.5.767

[B35] Li X Kulkarni RP Hill RJ Chamberlin HM HOM-C genes,Wnt signaling and axial patterning in the *C. elegans* posterior ventral epidermis Dev Biol 2009 332 1 156 165 10.1016/j.ydbio.2009.05.567 19481074

[B36] Studencka M Wesolowski R Opitz L Salinas-Riester G Wisniewski JR Jedrusik-Bode M Transcriptional repression of Hox genes by *C. elegans* HP1/HPL and H1/HIS-24 PLoS Genet 2012 8 9 e1002940 10.1371/journal.pgen.1002940 23028351 PMC3441639

[B37] Szabo E Hargitai B Regos A Tihanyi B Barna J Borsos E Takacs-Vellai K Vellai T TRA-1/GLI controls the expression of the Hox gene *lin-39* during *C. elegans* vulval development Dev Biol 2009 330 2 339 348 10.1016/j.ydbio.2009.04.005 19361495

[B38] Yu H Seah A Herman MA Ferguson EL Horvitz HR Sternberg PW Wnt and EGF pathways act together to induce *C. elegans* male hook development Dev Biol 2009 327 2 419 432 10.1016/j.ydbio.2008.12.023 19154732 PMC2695933

[B39] Zhao Z Boyle TJ Liu Z Murray JI Wood WB Waterston RH A negative regulatory loop between microRNA and Hox gene controls posterior identities in *Caenorhabditis elegans* PLoS Genet 2010 6 9 e1001089 10.1371/journal.pgen.1001089 20824072 PMC2932687

[B40] Niu W Lu ZJ Zhong M Sarov M Murray JI Brdlik CM Janette J Chen C Alves P Preston E Slightham C Jiang L Hyman AA Kim SK Waterston RH Gerstein M Snyder M Reinke V Snyder M Reinke V Diverse transcription factor binding features revealed by genome-wide ChIP-seq in *C. elegans* Genome Res 2011 21 2 245 254 10.1101/gr.114587.110 21177963 PMC3032928

[B41] Sulston JE Horvitz HR Post-embryonic cell lineages of the nematode. *Caenorhabditis elegans* Dev Biol 1977 56 1 110 156 10.1016/0012-1606(77)90158-0 838129

[B42] Sternberg PW Horvitz HR Pattern formation during vulval development in *C. elegans* Cell 1986 44 5 761 772 10.1016/0092-8674(86)90842-1 3753901

[B43] Greenwald I Riddle DL, Blumenthal T, Meyer BJ, Priess JR, Riddle DL, Blumenthal T, Meyer BJ, Priess JR Development of the Vulva C elegans II 1997 2 Cold Spring Harbor (NY): Cold Spring Harbor Laboratory Press

[B44] Sternberg PW Vulval development 2005 WormBook 1 28 10.1895/wormbook.1.6.1 PMC4781130 18050418

[B45] Clandinin TR Katz WS Sternberg PW *Caenorhabditis elegans* HOM-C genes regulate the response of vulval precursor cells to inductive signal Dev Biol 1997 182 1 150 161 10.1006/dbio.1996.8471 9073457

[B46] Wagmaister JA Gleason JE Eisenmann DM Transcriptional upregulation of the *C. elegans* Hox gene *lin-39* during vulval cell fate specification Mech Dev 2006 123 2 135 150 10.1016/j.mod.2005.11.003 16412617

[B47] Wagmaister JA Miley GR Morris CA Gleason JE Miller LM Kornfeld K Eisenmann DM Identification of cis-regulatory elements from the *C. elegans* Hox gene *lin-39* required for embryonic expression and for regulation by the transcription factors LIN-1, LIN-31 and LIN-39 Dev Biol 2006 297 2 550 565 10.1016/j.ydbio.2006.05.008 16782085

[B48] Guerry F Marti C Zhang Y Moroni P Jaquiéry E Müller F The Mi-2 nucleosome-remodeling protein LET-418 is targeted via LIN-1/ETS to the promoter of *lin-39*/Hox during vulval development in *C. elegans* Dev Biol 2007 306 2 469 479 10.1016/j.ydbio.2007.03.026 17466968

[B49] Grant K Hanna-Rose W Han M *sem-4* promotes vulval cell-fate determination in *Caenorhabditis elegans* through regulation of *lin-39* Hox Dev Biol 2000 224 2 496 506 10.1006/dbio.2000.9774 10926783

[B50] Chen Z Han M Role of *C. elegans* lin-40 MTA in vulval fate specification and morphogenesis Development 2001 128 23 4911 4921 11731470 10.1242/dev.128.23.4911

[B51] Kuntz SG Schwarz EM DeModena JA De Buysscher T Trout D Shizuya H Sternberg PW Wold BJ Multigenome DNA sequence conservation identifies Hox cis-regulatory elements Genome Res 2008 18 12 1955 1968 10.1101/gr.085472.108 18981268 PMC2593573

[B52] Reece-Hoyes JS Walhout AJ Gene-centered yeast one-hybrid assays Methods Mol Biol 2012 812 189 208 10.1007/978-1-61779-455-1_11 22218861 PMC3775493

[B53] Deplancke B Dupuy D Vidal M Walhout A A gateway-compatible yeast one-hybrid system Genome Res 2004 14 10B 2093 2101 10.1101/gr.2445504 15489331 PMC528925

[B54] Reece-Hoyes JS Diallo A Lajoie B Kent A Shrestha S Kadreppa S Pesyna C Dekker J Myers CL Walhout AJ Enhanced yeast one-hybrid assays for high-throughput gene-centered regulatory network mapping Nat Methods 2011 8 12 1059 1064 10.1038/nmeth.1748 22037705 PMC3235803

[B55] Koh K Peyrot SM Wood CG Wagmaister JA Maduro MF Eisenmann DM Rothman JH Cell fates and fusion in the *C. elegans* vulval primordium are regulated by the EGL-18 and ELT-6 GATA factors – apparent direct targets of the LIN-39 Hox protein Development 2002 129 22 5171 5180 12399309 10.1242/dev.129.22.5171

[B56] Walhout AJ Temple GF Brasch MA Hartley JL Lorson MA van den Heuvel S Vidal M GATEWAY recombinational cloning: application to the cloning of large numbers of open reading frames or ORFeomes Methods Enzymol 2000 328 575 592 11075367 10.1016/s0076-6879(00)28419-x

[B57] Yook K Harris TW Bieri T Cabunoc A Chan J Chen WJ Davis P de la Cruz N Duong A Fang R Ganesan U Grove C Howe K Kadam S Kishore R Lee R Li Y Muller HM Nakamura C Nash B Ozersky P Paulini M Raciti D Rangarajan A Schindelman G Shi X Schwarz EM Ann Tuli M Van Auken K Wang D WormBase 2012: more genomes, more data, new website Nucleic Acids Res 2012 40 Database issue D735 D741 22067452 10.1093/nar/gkr954 PMC3245152

[B58] Sluder AE Mathews SW Hough D Yin VP Maina CV The nuclear receptor superfamily has undergone extensive proliferation and diversification in nematodes Genome Res 1999 9 2 103 120 10022975

[B59] Deplancke B Mukhopadhyay A Ao W Elewa A Grove C Martinez N Sequerra R Doucette-Stamm L Reece-Hoyes J Hope I Tissenbaum HA Mango SE Walhout AJ A gene-centered *C. elegans* protein-DNA interaction network Cell 2006 125 6 1193 1205 10.1016/j.cell.2006.04.038 16777607

[B60] Zilliacus J Carlstedt-Duke J Gustafsson J Wright A Evolution of distinct DNA-binding specificities within the nuclear receptor family of transcription factors Proc Natl Acad Sci U S A 1994 91 10 4175 4179 10.1073/pnas.91.10.4175 8183888 PMC43747

[B61] Tucker M Sieber M Morphew M Han M The *Caenorhabditis elegans aristaless* orthologue, *alr-1*, is required for maintaining the functional and structural integrity of the amphid sensory organs Mol Biol Cell 2005 16 10 4695 4704 10.1091/mbc.E05-03-0205 16055504 PMC1237075

[B62] Melkman T Sengupta P Regulation of chemosensory and GABAergic motor neuron development by the *C. elegans* Aristaless/Arx homolog *alr-1* Development 2005 132 8 1935 1949 10.1242/dev.01788 15790968

[B63] Gerstein MB Lu ZJ Van Nostrand EL Cheng C Arshinoff BI Liu T Yip KY Robilotto R Rechtsteiner A Ikegami K Alves P Chateigner A Perry M Morris M Auerbach RK Feng X Leng J Vielle A Niu W Rhrissorrakrai K Agarwal A Alexander RP Barber G Brdlik CM Brennan J Brouillet JJ Carr A Cheung MS Clawson H Contrino S Integrative analysis of the *Caenorhabditis elegans* genome by the modENCODE project Science 2010 330 6012 1775 1787 10.1126/science.1196914 21177976 PMC3142569

[B64] Reece-Hoyes J Shingles J Dupuy D Grove C Walhout A Vidal M Hope I Insight into transcription factor gene duplication from *Caenorhabditis elegans* Promoterome-driven expression patterns BMC Genomics 2007 8 27 10.1186/1471-2164-8-27 17244357 PMC1785375

[B65] Labouesse M Hartwieg E Horvitz HR The *Caenorhabditis elegans* LIN-26 protein is required to specify and/or maintain all non-neuronal ectodermal cell fates Development 1996 122 9 2579 2588 8787733 10.1242/dev.122.9.2579

[B66] Labouesse M Sookhareea S Horvitz H The *Caenorhabditis elegans* gene *lin-26* is required to specify the fates of hypodermal cells and encodes a presumptive zinc-finger transcription factor Development 1994 120 9 2359 2368 7956818 10.1242/dev.120.9.2359

[B67] Andachi Y *Caenorhabditis elegans* T-box genes *tbx-9* and *tbx-8* are required for formation of hypodermis and body-wall muscle in embryogenesis Genes Cells 2004 9 4 331 344 10.1111/j.1356-9597.2004.00725.x 15066124

[B68] Inoue T Sternberg P *C. elegans* BED domain transcription factor BED-3 controls lineage-specific cell proliferation during organogenesis Dev Biol 2010 338 2 226 236 10.1016/j.ydbio.2009.12.005 20005870 PMC2862168

[B69] Ko LJ Engel JD DNA-binding specificities of the GATA transcription factor family Mol Cell Biol 1993 13 7 4011 4022 8321208 10.1128/mcb.13.7.4011-4022.1993 PMC359950

[B70] Koh K Rothman J ELT-5 and ELT-6 are required continuously to regulate epidermal seam cell differentiation and cell fusion in *C. elegans* Development 2001 128 15 2867 2880 11532911 10.1242/dev.128.15.2867

[B71] Gorrepati L Thompson KW Eisenmann DM *C. elegans* GATA factors EGL-18 and ELT-6 function downstream of Wnt signaling to maintain the progenitor fate during larval asymmetric divisions of the seam cells Development 2013 140 10 2093 2102 10.1242/dev.091124 23633508 PMC3640217

[B72] Quintin S Michaux G McMahon L Gansmuller A Labouesse M The *Caenorhabditis elegans* gene *lin-26* can trigger epithelial differentiation without conferring tissue specificity Dev Biol 2001 235 2 410 421 10.1006/dbio.2001.0294 11437447

[B73] Landmann F Quintin S Labouesse M Multiple regulatory elements with spatially and temporally distinct activities control the expression of the epithelial differentiation gene *lin-26* in *C. elegans* Dev Biol 2004 265 2 478 490 10.1016/j.ydbio.2003.09.009 14732406

[B74] Murray JI Boyle TJ Preston E Vafeados D Mericle B Weisdepp P Zhao Z Bao Z Boeck ME Waterston R Multidimensional regulation of gene expression in the *C. elegans* embryo Genome Res 2012 22 1282 1294 10.1101/gr.131920.111 22508763 PMC3396369

[B75] Open Biosystems [http://www.thermoscientificbio.com/openbiosystems]

[B76] Brenner S The genetics of *Caenorhabditis elegans* Genetics 1974 77 1 71 94 4366476 10.1093/genetics/77.1.71 PMC1213120

[B77] Riddle DL Blumenthal T Meyer BJ Priess JR C. elegans II 1997 Cold Spring Harbor, NY: Cold Spring Harbor Laboratory Press 21413221

[B78] Timmons L Fire A Specific interference by ingested dsRNA Nature 1998 395 6705 854 10.1038/27579 9804418

[B79] Image J [http://rsbweb.nih.gov/ij/]

[B80] Addgene [http://www.addgene.org]

[B81] Mohler WA Simske JS Williams-Masson EM Hardin JD White JG Dynamics and ultrastructure of developmental cell fusions in the *Caenorhabditis elegans* hypodermis Curr Biol 1998 8 19 1087 1090 10.1016/S0960-9822(98)70447-6 9768364

[B82] Pfaffl MW A new mathematical model for relative quantification in real-time RT-PCR Nucleic Acids Res 2001 29 9 e45 10.1093/nar/29.9.e45 11328886 PMC55695

[B83] Hoogewijs D Houthoofd K Matthijssens F Vandesompele J Vanfleteren JR Selection and validation of a set of reliable reference genes for quantitative sod gene expression analysis in *C. elegans* BMC Mol Biol 2008 9 9 10.1186/1471-2199-9-9 18211699 PMC2254638

[B84] Expression Patterns in Caenorhabditis [http://epic.gs.washington.edu]

