# Supplementary figures and images for: Multiple transcription factors directly regulate Hox gene lin-39 expression in ventral hypodermal cells of the C. elegans embryo and larva, including the hypodermal fate regulators LIN-26 and ELT-6
Source: BMC Dev Biol. 2014 May 13;14:17. doi: 10.1186/1471-213X-14-17 (PMC4051164; doi:10.1186/1471-213X-14-17)

Supplemental Figure 1

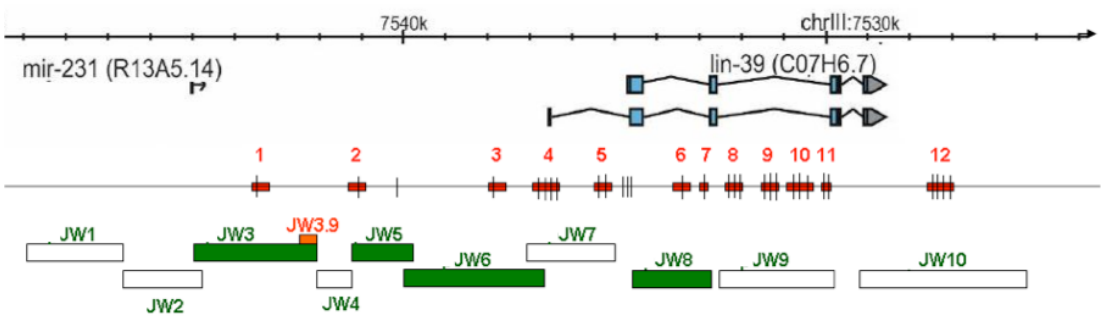

Supplement: Additional file 1: Figure S1 — lin-39 genomic region and fragments used in yeast one-hybrid screens. The top line shows 26 kb around the lin-39 locus with base locations on chromosome II shown. Two lin-39 transcripts and the upstream microRNA gene mir-231 are diagrammed below. JW1-10 (bottom) are lin-39 genomic regions previously used in reporter gene analysis; those in green drove GFP expression in vivo in lin-39 expressing cells [47]. pJW3.9 (orange box) is a 340 bp subfragment that drove GFP expression in P5 – P8 in the embryo [47]. Thirty-one evolutionarily-conserved regions (ECRs) with >75% identity in the lin-39 gene from three Caenorhabditis species were previously identified [47]. ECRs are shown on the middle line as unlabeled, vertical black lines. Twenty-seven ECRS were grouped into 12 PCR fragments (numbered 1–12; red boxes). The twelve fragments (YF1-12) and pJW3.9 were used as ‘baits’ in Y1H screens. [file 1471-213X-14-17-S1.pdf]

Supplemental Figure 2

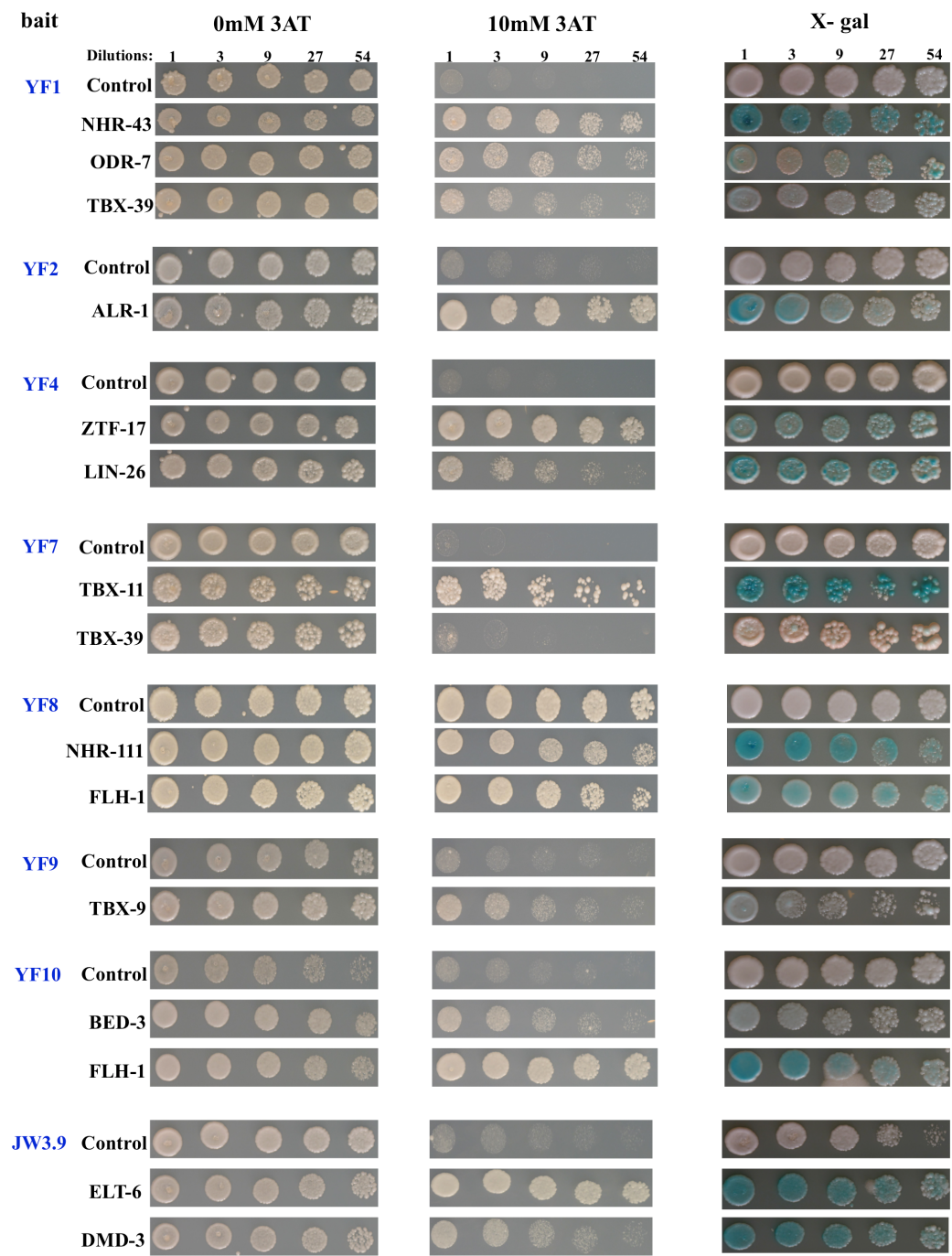

Supplement: Additional file 2: Figure S2 — Fourteen transcription factors interact with lin-39 genomic fragments in yeast one-hybrid assays (strain BY5444). Thirteen strains in background BY5444 containing fragments (YF1 - 12, pJW3.9) from the lin-39 genomic region were screened for interactions with C. elegans transcription factors by both the haploid library transformation method, and the robotically-assisted mating method (see Methods). Any positive interactions from primary screens were retested by retransformation of the rescued interacting plasmid back into the appropriate ‘bait’ strain (see Additional file 4: Table S2). Shown here are three-fold serial dilutions of each strain grown on SC–His-Ura-Trp plates with no 3AT (control plates), SC–His-Ura-Trp with 10 mM 3AT, and plates with X-gal. Bait strains transformed with the pDEST-AD empty vector were the ‘Control’. Positive interactions were considered those showing more growth on 3AT and/or more blue color on XGal than the control. Note that the YF8 strain shows considerable self-activation on 3AT, but positive interactions for NHR-111 and FLH-1 are observable on the XGal plates. [file 1471-213X-14-17-S2.pdf]

Supplemental Figure 3

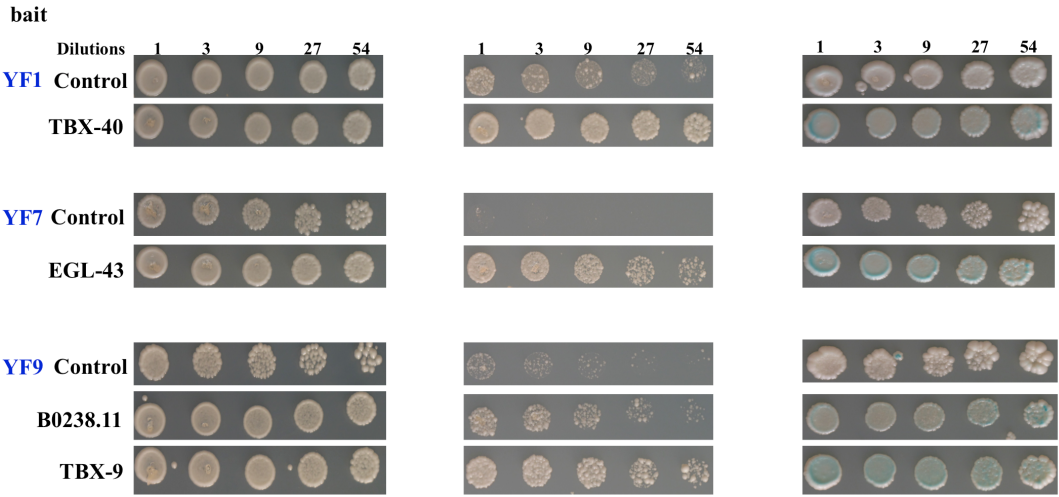

Supplement: Additional file 3: Figure S3 — Four transcription factors interact with lin-39 genomic fragments in yeast one-hybrid assays (strain YM4271). Thirteen strains in background YM4271 containing fragments (YF1 - 12, pJW3.9) from the lin-39 genomic region were screened for interactions with C. elegans transcription factors by the robotically-assisted mating method (see Methods). Positive interactions from the primary screen were retested by retransformation of the rescued interacting plasmid back into the appropriate ‘bait’ strain (see Additional file 4: Table S3). Shown here are three-fold serial dilutions of each strain grown on SC–His-Ura-Trp plates with no 3AT (control plates), SC–His-Ura-Trp with 10 mM 3AT, and plates with X-gal. Bait strains transformed with the pDEST-AD empty vector were the ‘Control’. Positive interactions were considered those showing more growth on 3AT and/or more blue color on XGal than the control. [file 1471-213X-14-17-S3.pdf]

Supplemental Figure 6

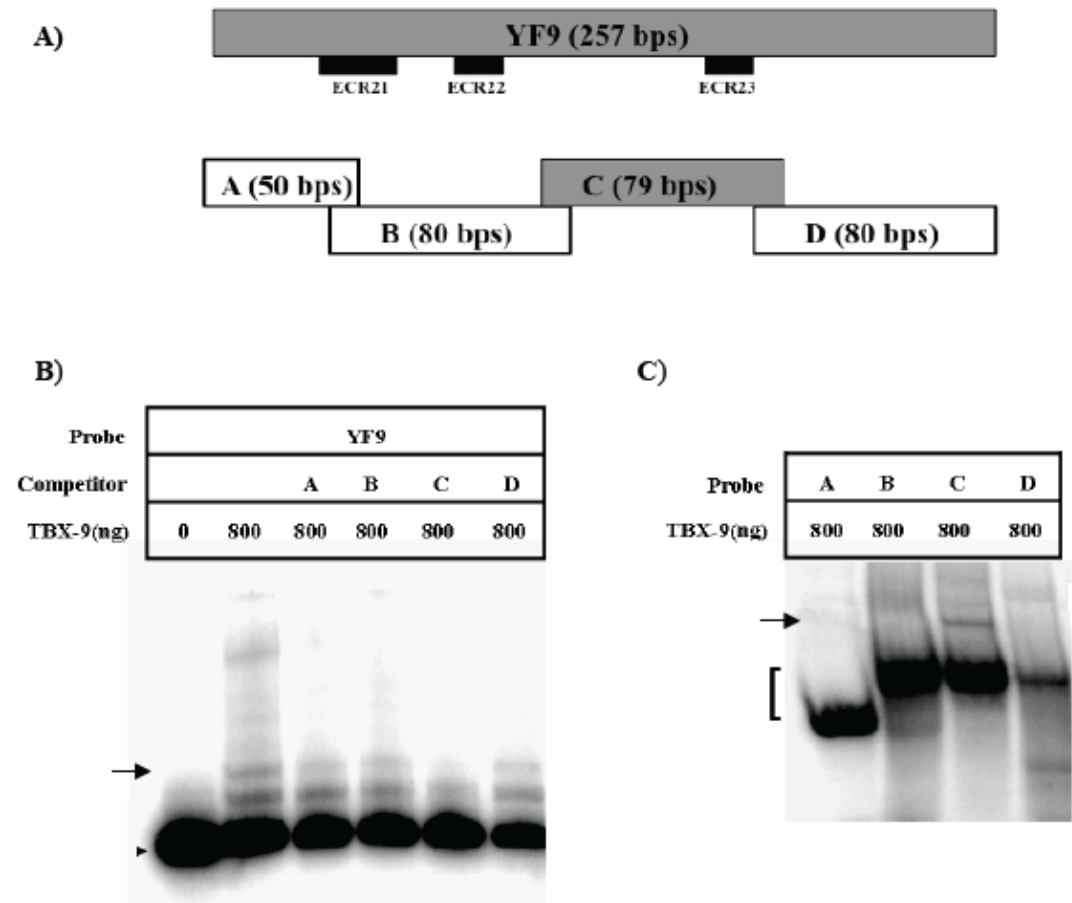

Supplement: Additional file 5: Figure S6 — TBX-9 binds a 79 bp region in fragment YF9. A) The top line shows fragment YF9 and the location of ECRs 21 – 23. Subfragments A – D are diagrammed below. Shading indicates the fragment bound by TBX-9 in vitro; B) Gel mobility shift assay with TBX-9 protein purified from E. coli and labeled fragment YF9 (lane 2) and competition with subfragments A – D (lanes 3–6). Arrowhead indicates free probe; arrow indicates the protein DNA complex that can be competed by fragment C. Two other bands appear with added TBX-9 protein: the upper band is competed away by all four competing fragments, while the lower band is competed by none of the fragments. The nature of these complexes is unknown, although they are likely to represent non-specific binding by TBX-9 or another protein. C) Gel mobility shift assay with TBX-9 protein and labeled fragment subfragments A – D. The bracket indicates the migration locations of free probes (which differ in size, see panel A). Arrow indicates a complex with TBX-9 and subfragment C. [file 1471-213X-14-17-S5.pdf]

Supplemental Figure 4

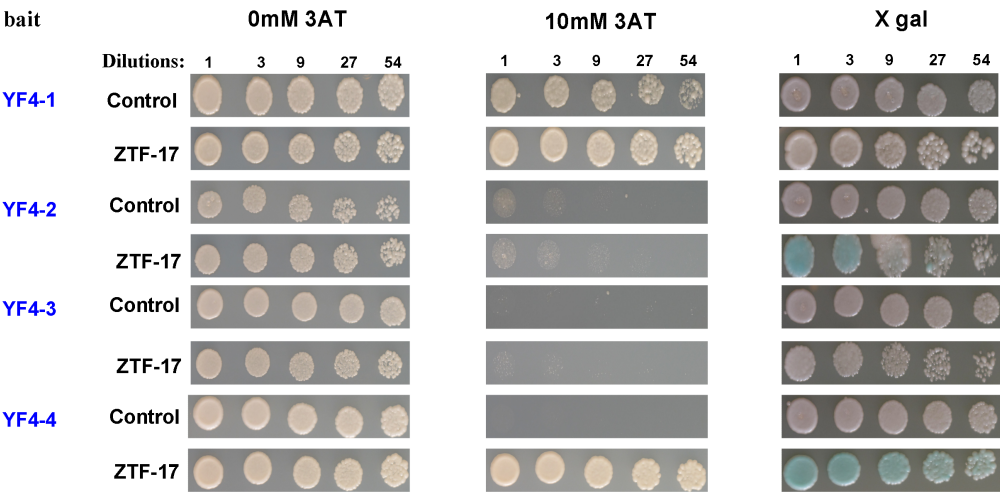

Supplement: Additional file 6: Figure S4 — ZTF-17 binds to fragment YF4-4 in yeast. BY5444 ‘bait’ strains containing subfragments of YF1 (YF4-1 to YF4-4; see Figure 1) were made and transformed with the plasmid encoding ZTF-17::Gal4AD or with the pDEST-AD empty vector as control. Three-fold serial dilutions of each strain grown on SC–His-Ura-Trp plates with no 3AT (control plates), SC–His-Ura-Trp with 10 mM 3AT, and plates with X-gal are shown. Fragment YF4-1 shows strong self-activation on 3AT. Only fragment YF4-4 shows a strong interaction with ZTF-17 on both 3AT and XGal plates. [file 1471-213X-14-17-S6.pdf]

Supplemental Figure 5

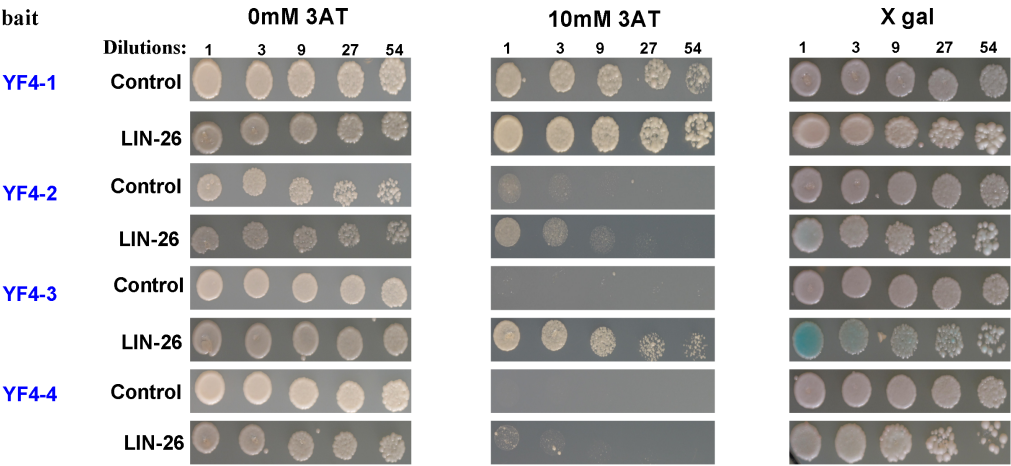

Supplement: Additional file 7: Figure S5 — LIN-26 binds to fragment YF4-3 using yeast. BY5444 ‘bait’ strains containing subfragments of YF1 (YF4-1 to YF4-4; see Figure 1) were made and transformed with the plasmid encoding LIN-26::Gal4AD or with the pDEST-AD empty vector as control. Three-fold serial dilutions of each strain grown on SC–His-Ura-Trp plates with no 3AT (control plates), SC–His-Ura-Trp with 10 mM 3AT, and plates with X-gal are shown. Fragment YF4-1 shows strong self-activation on 3AT. Only fragment YF4-3 shows a strong interaction with LIN-26 on both 3AT and XGal plates. [file 1471-213X-14-17-S7.pdf]

Supplemental Figure 7

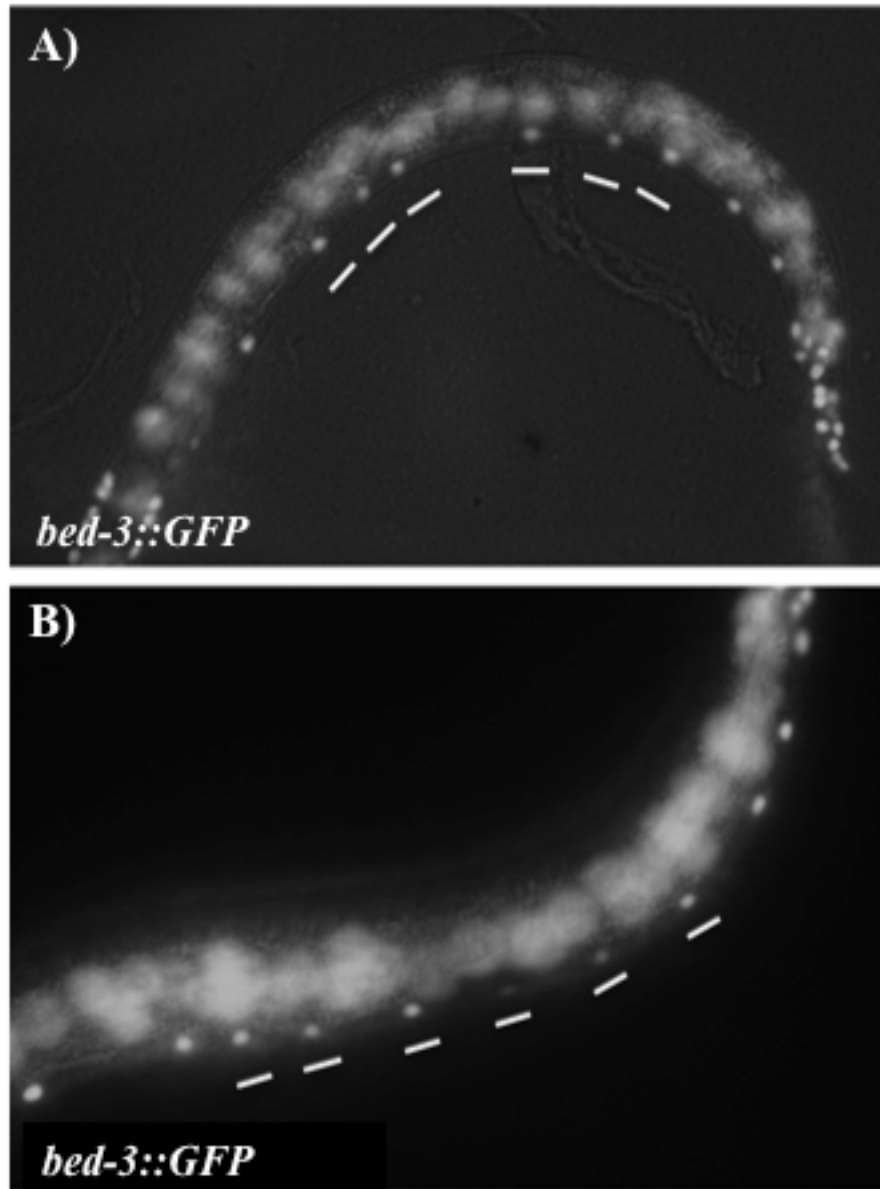

Supplement: Additional file 8: Figure S7 — bed-3::GFP expression in the larval VPCs. Expression from strains carrying syEx962 which contains the bed-3::GFP reporter pTI06.29 [68]. GFP expression is seen in the Pn.p cells in the L1 stage (A) and L2 stage (B), including the VPCs P3.p - P8.p (indicated by white bars). [file 1471-213X-14-17-S8.pdf]

Supplemental Figure 8

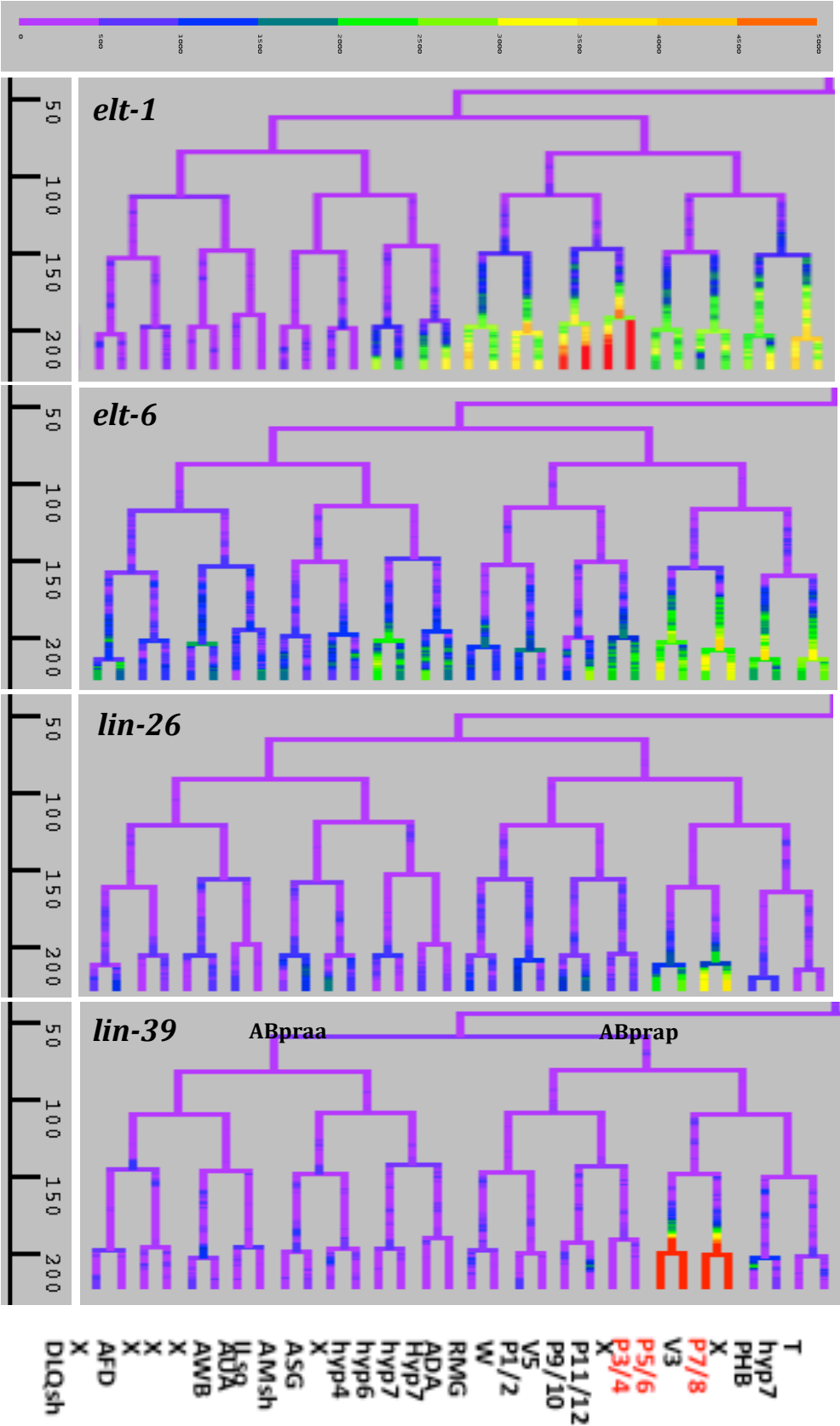

Supplement: Additional file 9: Figure S8 — Expression of transcription factor genes elt-1, elt-3, lin-26 and lin-39 in the ABpra lineage in developing embryos. The images shown are taken directly from the Expression Patterns in Caenorhabditis web site [84]. As described [74], live images were recorded from developing embryos that expressed a histone:mCherry fusion protein driven from the upstream promoter sequences for each of the indicated genes. Expression levels in individual embryonic cells were characterized and diagrammed on the cell lineage chart. An expression scale for all experiments is shown at the top and shows the fluorescence intensity of the reporter construct in the individual cells; time (in minutes) for each experiment is shown along the left. The data shown are from the following experiments: 20080128_elt-1_3.html, 20070817_elt-6_5.html, 20080805_lin-26_5_L1.html and 20071015_lin-39_9. [file 1471-213X-14-17-S9.pdf]
