# Supplementary material for: Multiple transcription factors directly regulate Hox gene lin-39 expression in ventral hypodermal cells of the C. elegans embryo and larva, including the hypodermal fate regulators LIN-26 and ELT-6
Source: BMC Dev Biol. 2014 May 13;14:17. doi: 10.1186/1471-213X-14-17 (PMC4051164; doi:10.1186/1471-213X-14-17)
Supplement: Additional file 4: Table S1 — Sequences of 12 YFs and pJW3.9. Table S2. Yeast one-hybrid screen with strain BY5444 (screen YM2). Table S3. Yeast one-hybrid screen with strain YM4271 (screen YM2). Table S4. Summary of yeast one-hybrid screen results. Table S5. Only GATA factor ELT-6 binds pJW3.9 in yeast. elegans GATA factors. Table S6. Oligonucleotides for EMSA probes. [file 1471-213X-14-17-S4.docx]

**Additional file 4**

**Table S1. Sequences of 12 YFs and pJW3.9**

| **Fragment (contained ECR)** | **sequence** |
| --- | --- |
| **YF1(ECR1)** | **aggccacatcctcccatttgatatgcatacttgactcaaatcaggagatacaggaaaacaatagacttctcgaaaggcgtgtacaactacattttattctattgatatgcaaattaacaagatgaagatgggggaatattgaataaaattggcacttcatttggctacgtttttgatatttatttatctacgattttgtaaaatggttagaataagtattgtctgttaagcatttcagactgcttcaacttttgatcaatttgtgatcatttcaaacaatttgtaagttttttgttctttggatttataaattggcacaatcgagaactttttgcatgttc** |
| **YF2(ECR2)** | **cgttgttcatttgcatgttcaggggttactgtaacatttgttgttctgcccccatatgcttcctgctgtgtgtggcacgagcgccaataatagatgtgtctggctttgaaaagaatgccctatatgcaatcgatacttctagaatccctttgaataaatgattctctggggttgaaattccagttcttgagattgttTAAGTTTTGGTAGTTTTTAATtcttgaaatttgcgtccaggttagtctactactatgatatgtctttaaattggagacttttaaaattcggcagatttccaaacaaaactacgc** |
| **YF3(ECR4)** | **cgtttctgtttgcactagattggaagaccaaaaaaaatcaaaatgaggaattgtcgaacatcaatttgtaaaagagagaagatccctacacttcttcaacctattttttcttgattcgtgtacctacaaatgagtaattggatgccattcattccacacttcttgatgcttcctgacactcataaatcactgtaaaagagacaggaaatagacttgagtcaattgcataatcacttactaatcatttactcac** |
| **YF4(ECR7-10)** | **cgactgactcttgacaacttgtccaatgggaaaaagttggtcaagattttgttcctaaaagcggcaacttaaaagcgaaaattcattctgctcaactcttctactcttggagcccaaaagcttacggtacctgtcgttctcaccccatgtccatccagcgctgtcagtgatgaattgaaaaggcgagagcaagagatgtgctctcggctccacgaacctctttgatctatgccgacagtgggggagattggagaagaagcatgctccacccttcacgtgtatcagtacgtctgcgtcttcttgtgcgtgccgcccggattagaagacatgaagtgggagggcgatagaggaaattccgatggcaataccaac** |
| **YF5(ECR11,12)** | **ccatacttcttcttgacctcatagattgcttactagtttatataaagctatttagagcaattatccaatcttataaggtaaaacccgcgggtcttaaaccatgaccacccacttgagcacacccacaaaaacaattcgtctctcgcagattgctcctcaatttatcaccatcatcttttctctctccatttccaactctttacttttcttctctttttgttctgtttccgttcttaagcttttgttgttttcctagttttaatgatttcctaaggattaggtacaagtagttgatagg** |
| **YF6(ECR16)** | **cgcctcccactggtttgaaacttctgtcatttcccgccaaaaattgaaaatcaatcaattcctcagcgtttctgaaaataatctgacaacgcgatcaattttattttctcttttgaagtgagtggactggcgcctggtgactaccattggaagagtaggaattgaaataaaatttatgagaacgagttacaaaatggagaataagaggaagaagagtattcagattgagttaatgatcgttccatcgatttttgtacttttttagattcacaattggaagctgcaaaaacctccaagcactgg** |
| **YF7(ECR17)** | **gtaggaaaaccaaacgcagtgagggaaaatgaaagaagaagactgaagagagtgaaagagtgagaggaagagtttcaagattaattgttcccgctgtttgtctcttcttggatgctaagatgtatcggttagagaatctcttccaaaatcttcgcaac** |
| **YF8(ECR18-20)** | **gacctttaatggcaatttgtttgggtaattataaatcaatttactactgaatagtggaaggaagctacagtactccccttcttttccctctctccacatcattttgacatgaagcataaggaagaaggcgacgcgcggcgccctctctctcgatagttgacgtgaaagcagagctcgaccccaagaatagtcagtggcggagcaaaaagagagatggcatgtcgcgagcccatttatctttcgtgtcaagaccccccgccactcactttctgtcgtgtggtgctggttcctaaccacttcaagcccaaacgatggtgtgtatgtgtcattg** |
| **YF9(ECR21-23)** | **gttacaccgtgttctccctcatcacagctatcagagcaacttgtcaaagtatttggactccccgcagcggtatctctcttattgacagttgatttgggtgtgcaccttgcgttcggacagcattgtgcactttgtgttatgtcttgcggcttcttttctactcttgacacgcttgtgatcgatgatgatgattcggattctagtggtgaagcactgatggaggaaattaacaagttgggcaagagtggaggttatcg** |
| **YF10(ECR24-26)** | **ctgagcgacagaataattacgaatcctcgaaacccatacatggaaaccgattttcccgattaaagtctcacgcgtgtctcaaaatgagagacaagctgtctgttggaccaacgcacaggtgtcttcagcaacattgtctttttctctctcttttctttcccatgtcggccatatggcgcagcgccccgtcagaaaccgaccggcgtctttagcacaacatccattatatgtgaatagttgaaatgaaaatgaacgttgaaaatgaattgaatctattgactcgtttatttctcgagatatttatggggatcgagaacag** |
| **YF11(ECR27,28)** | **ctctccagttgttttgttttggcacccctatcataaattgctcaacaaacagtcagctgtttggcaagaggtgacaatctttacaactgatatgttctatatattcgcaaggattgtcaattgtatattatgatggcgatctccttccgaagagtctctctcggccctaaagtgtgacaagtgtgaaaacgatgaccctcctcggggagagggccccgcgtgcgcgcaccctcatgtccatcctcgaaattctg** |
| **YF12(ECR29-33)** | **gttgctccagaatatcgggtttcgccaaaactgcaatccagaactaaatcaaaccagtttagaccttgaccgagcataaactaattttatctacttataagactttccaacaaaacctataagacaatctatcacctcttcgatcaatgtttacacccgcgtgggtccatctgacctcttctttcgctcccgaaaaacagcaattagtctcatcgtgtcggcttctctttcctacttaccactattaactgtaaaacaattaatatatgctgacagcagaagcgcccacatacaactcaatatctttaatctagccgatgaatttattggattatgtatgtgctccaaggcacgactacggtaggtttgaaatattcgaagagagagataggaagatccgatgggagcgaccaatgagagatgaaatgagggaggagtctttgtatagttaagta** |
| **JW3.9** | **catagaaaatcagcgtgtgcctttaaacttaaatttctgtacaatcactatatccagcaaccagcacaaccctatcctttctggtcctaatttatcaatttatctttcttgttatcatatctcaaattccatcaaaatagtgtttcttttgatattaccgacatcgtaatgtatcattttatcgcaattagtcatgttatcacacgggaaggtgttactattatcagttcctcatttgttccatttgttgctgttactttttctttatccagtttttaatgtgctttaaattatatttggggtttattttaccattttcatatttgaaggtcttgagg** |

The sequences of the fragments used in the yeast one-hybrid assays (YFs) are shown, with evolutionarily conserved regions (ECRs) highlighted in red.

**Table S2. Yeast one-hybrid screen with strain BY5444 (screen YM2).**

| **BAIT** | **BAIT Self Activity** | **gene** | **sequence** | **readout strength** |
| --- | --- | --- | --- | --- |
| **YF1** | LOW | ***odr-7*** | **T18D3.2** | STRONG |
|  |  | ***tbx-39*** | **Y73F8A.16** | STRONG |
| **YF2** | LOW | *none* |  |  |
| **YF3** | LOW | *tab-1* | F31E8.3 | STRONG |
|  |  | *ets-5* | C42D8.4 | WEAK |
| **YF4** | LOW | *hlh-25* | C17C3.7 | STRONG |
|  |  | ***lin-26*** | **F18A1.2** | STRONG |
| **YF5** | LOW | *sptf-3* | Y40B1A.4 | MEDIUM |
|  | LOW | *odr-7* | T18D3.2 | WEAK |
| **YF6** | LOW | *ceh-38* | F22D3.1b | STRONG |
| **YF7** | LOW | ***tbx-11*** | ***F40H6.4*** | WEAK |
|  |  | ***tbx-39*** | ***Y73F8A.16*** | STRONG |
| **YF8** | MED | *T22E7.2* | *T22E7.2* | STRONG |
|  |  | ***flh-1*** | ***Y11D7A.12ab*** | STRONG |
|  |  | ***nhr-111*** | ***F44G3.9*** | STRONG |
|  |  | *T20F10.2* | *T20F10.2* | WEAK |
|  |  | *nhr-65* | *Y17D7A.3ab* | WEAK |
|  |  | *C43H6.7* | *C43H6.7* | WEAK |
|  |  | *R13F6.5* | *R13F6.5* | WEAK |
|  |  | *M18.8* | *M18.8* | WEAK |
| **YF9** | LOW | ***tbx-9*** | ***T07C4.6*** | STRONG |
|  |  | *tbx-8* | *T07C4.2* | STRONG |
| **YF10** | LOW | ***bed-3*** | ***F25H8.6*** | STRONG |
|  |  | ***flh-1*** | ***Y11D7A.12ab*** | STRONG |
|  |  | *hlh-27* | *C17C3.10* | STRONG |
| **YF11** | HIGH | *tbx-9* | *T07C4.6* | STRONG |
|  |  | *tbx-37* | *Y47D3A.12* | STRONG |
|  |  | *tbx-8* | *T07C4.2* | STRONG |
|  |  | *tbx-38* | *C24H11.3* | STRONG |
| **YF12** | LOW | *none* |  |  |
| **JW3.9** | LOW | *tbx-9* | *T07C4.6* | STRONG |
|  |  | ***dmd-3*** | ***Y43F8C.10*** | STRONG |
|  |  | *tbx-8* | *T07C4.2* | STRONG |
|  |  | ***elt-6*** | ***F52C12.5*** | STRONG |

Thirteen ‘bait’ strains (column 1) in parent strain BY5444 were tested for self-activation (column 2 ; LOW’, ‘MED’, and ‘HIGH’ represent the degree of the colony growth on 3AT plates and the intensity of blue on X-gal plates, observed by eye). All 13 strains were used in robotically-assisted yeast mating assays. Thirty-two interactions (column 3, sequence name column 4) were initially identified based on ‘readout strength’ (column 5). ‘WEAK’, ‘MEDIUM’, and STRONG represent colony growth on 3AT plates and the intensity of blue on X-gal plates compared to a control strain, observed by eye. All plasmids were rescued from yeast and retransformed back into the appropriate bait strains manually; those factors that repeated after manual retransformation are shown in bold (see Supplemental Figure 2).

**Table S3. Yeast one-hybrid screen with strain YM4271 (screen YM2).**

| **BAIT** | **BAIT Self Activity** | **gene** | **sequence** | **readout strength** |
| --- | --- | --- | --- | --- |
| **YF1** | low | ***tbx-40*** | ***Y73F8A.17*** | MEDIUM |
| **YF2** | low | *none* |  |  |
| **YF3** | low | *none* |  |  |
| **YF4** | low | *none* |  |  |
| **YF5** | low | *none* |  |  |
| **YF6** | low | *mig-5* | *T05C12.6* | STRONG |
| **YF7** | low | ***egl-43*** | ***R53.3*** | WEAK |
| **YF8** | high | *none* |  |  |
| **YF9** | low | ***B0238.11*** | ***B0238.11*** | MEDIUM |
|  |  | ***tbx-9*** | ***T07C4.6*** | STRONG |
| **YF10** | low | *bed-3* | *F25H8.6* | MEDIUM |
|  |  | *mig-5* | *T05C12.6* | MEDIUM |
|  |  | *ztf-15* | *R06C7.9* | WEAK |
|  |  | *hlh-27* | *C17C3.10* | STRONG |
| **YF11** | high | *none* |  |  |
| **YF12** | low | *none* |  |  |
| **JW3.9** | low | *elt-7* | *C18G1.2* | WEAK |

Thirteen ‘bait’ strains (column 1) in parent strain YM4271 were tested for self-activation (column 2 ; LOW’, ‘MED’, and ‘HIGH’ represent the degree of colony growth on 3AT plates and the intensity of blue on X-gal plates, observed by eye). All 13 strains were used in robotically-assisted yeast mating assays. Ten interactions (gene name column 3, sequence name column 4) were initially identified based on ‘readout strength’ (column 5). ‘WEAK’, ‘MEDIUM’, and STRONG represent colony growth on 3AT plates and the intensity of blue on X-gal plates compared to a control strain, observed by eye. All plasmids were rescued from yeast and retransformed into the appropriate bait strains manually; those factors that repeated after manual retransformation are shown in bold (see Supplemental Figure 3).

**Table S4. Summary of yeast one-hybrid screen results.**

| **BAIT** | **HT1** | **DM2** | **HT2** | **DM3** | **HT3** |
| --- | --- | --- | --- | --- | --- |
| **YF1** | ***nhr-43*** | *odr-7, tbx-39* | *odr-7, tbx-39* | *tbx-40* | *tbx-40* |
| **YF2** | ***alr-1*** |  |  |  |  |
| **YF3** |  | *tab-1, ets-5* |  |  |  |
| **YF4** | ***ztf-17*** | *hlh-25,* ***lin-26*** | ***lin-26*** |  |  |
| **YF5** |  | *sptf-3 ,odr-7* |  |  |  |
| **YF6** |  | *ceh-38* |  | *mig-5* |  |
| **YF7** |  | *tbx-11, tbx-39* | *tbx-11, tbx-39* | *egl-43* | *egl-43* |
| **YF8** |  | *flh-1, nhr-111, T22E7.2, nhr-65, T20F10.22, C43H6.7, M18.8, R13F6.5* | *flh-1, nhr-111* |  |  |
| **YF9** |  | *tbx-8,* ***tbx-9*** | ***tbx-9*** | ***tbx-9****, B0238.11* | ***tbx-9****, B0238.11* |
| **YF10** |  | ***bed-3****, flh-1, hlh-27* | ***bed-3****, flh-1* | ***bed-3****, mig-5, ztf-15, hlh-27* | auto-activate |
| **YF11** |  | *tbx-8, tbx-9, tbx-37, tbx-39* |  |  |  |
| **YF12** |  |  |  |  |  |
| **JW3.9** |  | *dmd-3,* ***elt-6,***  *tbx-8, tbx-9,* | *dmd-3,* ***elt-6*** | *elt-7* |  |

Column 1 shows each bait strain used. Column 2 shows three factors identified in the haploid library transformation screens (HT1). Column 3 shows the initial 32 interactions detected by yeast mating screens using BY5444 strains (DM2), and column 4 shows those 12 interactions that repeated by haploid transformation assay (HT2). Column 5 shows the initial 10 interactions detected by yeast mating screens using YM4271 strains (DM3), and column 6 shows the four interactions that repeated by haploid transformation assay (HT3). Genes in bold are those that were characterized further in the text.

**Table S5. Only GATA factor ELT-6 binds JW3.9 in yeast*. elegans* GATA factors.**

|  | **3AT (mM)** | **X-gal** |
| --- | --- | --- |
| ***control*** | **0** | **white** |
| ***elt-1*** | **0** | **white** |
| ***elt-2*** | **0** | **white** |
| ***elt-3*** | **0** | **white** |
| ***egl-18*** | **0** | **white** |
| ***elt-6*** | **20** | **blue** |
| ***elt-7*** | **0** | **white** |
| ***end-1*** | **0** | **white** |
| ***end-3*** | **0** | **white** |
| ***med-2*** | **0** | **white** |

The JW3.9 bait strain was transformed individually with nine plasmids containing individual GATA factor ORFs fused to the GAL4 activation domain (control = pDEST-AD vector). Only ELT-6 shows a positive interaction with JW3.9 based on growth above background on 3AT plates and blue color on XGal plates.

**Table S6. Oligonucleotides for EMSA probes.**

**A.**

| **Probe larger than 100 bp (PCR primers)** | | | |
| --- | --- | --- | --- |
| **Fragment** | **size (bp)** |  | **primer sequence** |
| **YF1** | 342 | FW | caggccacatcctcccatttgatatgc |
|  |  | RV | gaacatgcaaaaagttctcgattgtgcc |
| **YF1 (Mut)** | 342 | FW | caggccacatcctcccatttgatatgcatact**gtga**tcaaat |
|  |  | RV | gaacatgcaaaaagttctcgattgtgcc |
| **YF4-2** | 186 | FW | gagcaagagatgtgctctcgg |
|  |  | RV | gttggtattgccatcggaattt |
| **YF4-3** | 103 | FW | gagcaagagatgtgctctcgg |
|  |  | RV | cgtactgatacacgtgaaggg |
| **YF4-4** | 110 | FW | gctccacccttcacgtgtatc |
|  |  | RV | gttggtattgccatcggaattt |

**B.**

| **Probe less than 100 bp** | | |
| --- | --- | --- |
| **Fragment** | **size (bp)** | **sequence** |
| **ECR2** | 40 | cccatatg*cttcctgctgtgtgtggcacgag*cgccaataa |
| **YF4-3-1** | 51 | gagcaagagatgtgctctcggctccacgaacctctttgatctatgccgaca |
| **YF4-3-2** | 52 | gtgggggagattggagaagaagcatgctccacccttcacgtgtatcagtacg |
| **S1** | 40 | attttatc*gcaattagtcatgttatcacacg*ggaaggtgt |
| **S1M1** | 40 | attttatc*gcaattagtcatg****ggtacc****cacg*ggaaggtgt |
| **S1M2** | 40 | att**ggtac*c****caattagtcatgggtacccacg*ggaaggtgt |

A) EMSA probes larger than 100 bp were generated by PCR using the primers listed. The sequence mutated for the YF1 mutant probe is indicated in bold. B) EMSA probes smaller than 100 bp were made by hybridization of complementary oligonucleotides; the sequence of one primer is shown. ECR2 and S1 sequences are shown in italics. S1M1 and S1M2 contain S1 with mutations in one or two GATA sites respectively (altered GATA sites shown in bold).
